# Intracellular selection of *trans*-cleaving hammerhead ribozymes

**DOI:** 10.1093/nar/gkz018

**Published:** 2019-01-16

**Authors:** Xin Huang, Yongyun Zhao, Qinlin Pu, Getong Liu, Yan Peng, Fei Wang, Gangyi Chen, Meiling Sun, Feng Du, Juan Dong, Xin Cui, Zhuo Tang, Xianming Mo

**Affiliations:** 1 Natural Products Research Center, Chengdu Institution of Biology, Chinese Academy of Science, Chengdu 610041, P.R. China; 2 Laboratory of Stem Cell Biology, State Key Laboratory of Biotherapy, West China Hospital, Sichuan University, Chengdu 610041, P.R. China

## Abstract

Hammerhead ribozyme is the smallest and best characterized catalytic RNA-cleaving ribozyme. It has been reported as potential therapeutic tools to manipulate the expression of target genes. However, most of naturally occurring hammerhead ribozymes process self-cleavage rather than cleave substrate RNA *in trans*, and its high intracellular activity relies on the tertiary interaction of Loop II and steam I bulge, resulting in decreased performance as applied in gene silencing. We described a direct intracellular selection method to evolve hammerhead variants based on *trans-*cleavage mode *via* using a toxin gene as the reporter. And a dual fluorescence proteins system has also been established to quantitatively evaluate the efficiency of selected ribozymes in the cell. Based on this selection strategy, we obtained three mutants with enhanced intracellular cleaving activity compared to wide type hammerhead ribozyme. The best one, TX-2 was revealed to possess better and consistent gene knockdown ability at different positions on diverse targeted mRNA either in prokaryotic or eukaryotic cells than wild-type hammerhead ribozyme. These observations imply the efficiency of the intracellular selection method of the *trans*-acting ribozyme and the potentials of improved ribozyme variants for research and therapeutic purposes.

## INTRODUCTION

Catalytic RNAs were termed ribozymes because of their similar catalytic property to protein enzymes. The hammerhead ribozyme (HHRz), the smallest and best-characterized self-cleaving ribozyme (1,2) (Supplementary Figure S1), was originally discovered in infectious satellite RNA (3). Based on the unique secondary structure, hammerhead ribozymes have been engineered to cleave any given RNA by an intermolecular attack (*trans*-cleavage), through recognizing substrate by two binding arms and cleaving adjacent to the sequence NUX**↓** (N is any base and X is A, C or U) (4). Although small ribozymes, such as Hairpin or hepatitis delta virus (HDV), have *trans*-cleaving potential to be applied in the *trans*-cleavage of targeted mRNA for suppression of gene expression (5,6), HHRz is the most attractive candidate for the development of ribozyme-based therapeutic tools because of its small size and flexibility in design (7–12). The most important difference between ribozyme technologies and siRNA tools is that siRNAs require the recruitment of endogenous proteins to achieve high intracellular activities. Although HHRz appears slightly less effective than siRNAs, they offer advantages due to their specificity of target recognition and precise cleavage, without any reported ‘off-target’ effects (13). As the first gene therapy agent to reach clinical trials, HHRz remains the only reported agent tested in a randomized, double-blind cell-delivered clinical phase II trial for HIV-1 infection (14). The study indicates that gene therapy based on HHRz is safe and biologically active in individuals with HIV-1. However, to improve the antiviral efficacy in future clinical trials, the identification of new ribozymes and RNA conjugation strategies is required (15).

**Figure 1.**
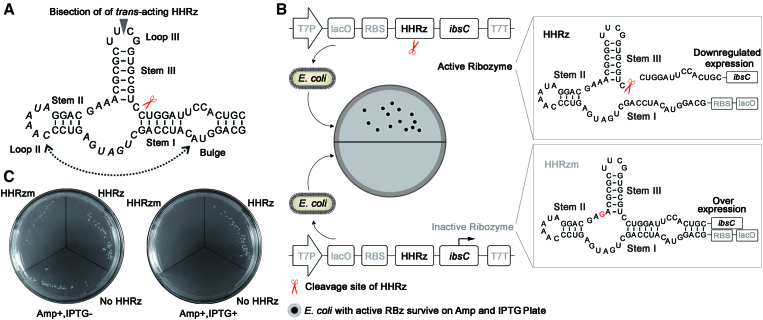
Strategy of direct intracellular selection of HHRz. (**A**) Secondary structure of the hammerhead ribozyme. The cleaving site is identified by a red scissor, and the triplet next to the cleavage site is labeled in red. Bisection of *trans*-acting is displayed by a gray arrow. The randomized regions used in the study is labeled by italic. (**B**) Schematic diagrams of intracellular cleavage of *cis*-acting HHRz. HHRzs were inserted into upstream of *ibsC* to regulate its expression. Active HHRz could cleave the mRNA of *ibsC* to render for the normal growth of *E. coli* on resistant plate, while cells with inactive HHRz could not survive on the plate due to the toxin expression in the induction of IPTG. (**C**) Intracellular regulation of IbsC toxin by active HHRz and inactive HHRzm. Only *E. coli* transformed by WT-HHRz could yield bacterial colony on selection culture plate due to inherent cleavage capacity of HHRz.

Hammerhead ribozymes catalyze self-cleavage rather than cleave substrate RNA *in trans*. Moreover, the loop-loop interaction in hammerhead pseudoknot tertiary structure has been proved to be very important for the *cis-*cleavage in the cell (Figure 1A, Supplementary Figure S1) (2). The tertiary interactions of HHRz induce the structural organization of the catalytic core and dramatically increase its activity, promoting efficient self-cleave of HHRz in physiological low Mg^2+^ concentrations (16–19). However, to knock down the expression of a given gene, the *trans*-acting ribozyme binds to the targeted mRNA molecule through perfectly-matched duplex. The loss of the loop-loop interaction will affect the folding and activity of ribozyme, thus causing the performance degradation of engineered HHRz in gene therapy.

Several selection attempts *in vitro* have been made to overcome the problem, including optimizing the catalytic core sequences (20) and stem-loop II (21,22), as well as diversifying the cleavage sites (23–25), obtaining HHRz variants working in low Mg^2+^ concentration (21,26–28). However, the cleavage activities of ribozymes *in vitro* are not necessarily correlated with their performance in the cellular environment, where various components and compartments probably affect the diffusion, folding, and binding of HHRzs. *In vivo* selection method has been established to evolve functional *trans*-splicing group I intron (29). To obtain new HHRz variants that function in physiological conditions, a hybrid *in vitro/in vivo* selection scheme has been developed, in which randomized HHRzs were transcribed in cell and the functional species were amplified with PCR *in vitro* (30,31). It is important to note that all those attempts to evolve HHRzs were based on the *cis*-cleavage mode, while the HHRzs applied in gene therapy catalyze the cleavage of RNA in *trans* mode. In this study, we describe a completely direct intracellular selection method to evolve HHRz variants based on *trans-*cleavage mode *via* using a toxin gene as the reporter. And a dual fluorescence proteins system has also been established to quantitatively evaluate the efficiency of selected HHRzs in the cell. Through these methods, novel HHRz variants for *trans*-cleavage have been obtained, which revealed the improved catalytic ability to modulate gene expressions in the cell.

## MATERIALS AND METHODS

### Cells and reagents

High fidelity restriction endonuclease *Bam*HI, *Eco*RI, *Hin*dIII, *Sgr*AI, *Xba*I, *Xho*I, *Sal*I, *Blp*I, *Nhe*I and T4 ligase were purchased from NEB (New England Biolabs, MA, USA). T7 RNA polymerase, FastAP and PNK was purchased from Formentas (Fermentas, Thermo Fisher Scientific, USA). Taq DNA polymerase and PFU DNA polymerase were purchased from TransGen (TransGen Biotech, Beijing, China). Bacterial Strain JM109(DE3) and Pro 5-alpha were purchased from Promega (Promega, WI, USA). BL21(DE3) were purchased from TransGen. pET32 and pET28 were purchased from Novagen (Merck, Darmstadt, Germany). pmCherry-C1 was gift from professor Hu Qinxue's laboratory. Plasmids were prepared by AxyPrep^TM^ Plasmid Miniprep Kit (Axygen, Corning, MA, USA). DNA products were purified by AxyPrep^TM^ PCR clean up Kit (Axygen). Restriction enzyme digested fragments were extracted by AxyPrep^TM^ DNA Gel Extraction Kit (Axygen). Rabbit monoclonal to GAPDH (ab128915), rabbit monoclonal to Bcl-2 (ab32124) and rabbit monoclonal to β-Actin (ab115777) were purchased from Abcam (Abcam, Cambridge, UK) and HRP-conjugated Goat Anti-Rabbit secondary antibody was purchased from BBI (BBI, Sangon Biotech, Shanghai, China). Hela and MDA-MB-231 was purchased from ATCC (American Type Culture Collection, VA, USA).

### Library design and synthesis of random single-strand DNA sequence

The DNA sequences of the selection pools for *trans*-acting HHRz were as follow: R1, 5′-AACGAATTCCATGAATCCAG(N7)GTCCCAAATAGGACGAAACGCACCGAATCTAATACGGCCGCGGGTCCAGGGTTCAAGTCCCTGTTCGGGCGCCAGGATCCTCG-3′. R2, 5′-AACGAATTCCATGAATCCAGCTGATGAGTCC(N6)GGACGAAACGCACCGAATCTAATACGGCCGCGGGTCCAGGGTTCAAGTCCCTGTTCGGGCGCCAGGATCCTCG-3′. N7 and N6 were seven or six fully random nucleotides in long catalytic core and loop II of HHRz respectively. 5′-end of HHRz was restriction enzymes site (*Eco*RI), binding sequence of substrate and upstream linker connecting HHRz to tRNA; 3′-end of HHRz was downstream tRNA sequence, binding sequence of substrate and restriction enzymes site (*Bam*HI). The selection dsDNA pools were prepared from fusion PCR products of ai-HR1/ai-HR2, ai-H2 and R-SR54 (Supplementary Table S1). Fusion PCRs were performed as follows: pre-denaturation at 95°C for 1 min, then 10 cycles (30 s at 95°C, 30 s at 51°C, 40 s at 72°C of each cycle).

### Intracellular selection of *trans*-cleaving HHRz by toxin protein

Vector construction of *trans*-cleaving HHRz was described in Supplementary Figure S6–S9. DNA products were purified by AxyPrep^TM^ PCR clean up Kit (Axygen) and double digested by *Eco*RI and *Bam*HI (NEB) in 37°C for 3 h. Digested fragments of DNA pools were purified by AxyPrep^TM^ PCR clean up Kit (Axygen) again. DNA fragments of vector pTR7a1Gmai (Supplementary Table S2) digested by same restriction enzyme were extracted by AxyPrep^TM^ DNA Gel Extraction Kit (Axygen). Digested products were ligated by NEB T4 ligase in 4°C overnight giving raise of recombinant plasmid pools for intracellular selection. The presence of saline ions would decrease the transformed efficiency of recombinant vectors. The ligation products were extracted once with an equal volume of phenol-chloroform extraction and then ethanol precipitated following a recovery in 10μl sterile deionized water.

Electrotransformed competent cells were prepared according to the manufactories' protocol (Bio-Rad) with moderate modifications. Purified ligation products were electrotransformed into bacterial expression strain BL21(DE3) and recovered in 37°C and 150 rpm for 1 h. All cells were harvested by centrifugation in 4°C and 4000 rpm for 5 min following coating on ampicillin resistant culture plate. In the induction of IPTG (isopropyl β-d-thiogalactoside), cells with potential active HHRz mutants would survive on the plate and be identified by DNA sequencing.

### Definition of *trans*-acting mutants based on dual fluorescent proteins using flow cytometry (FCM)

Recombinant vectors containing HHRz, HHRzm and selected variants were electrotransformed into bacterial expression strain BL21(DE3) and recovered in 37°C and 150 rpm for 1 h. 50 μl of transformed cells were coated on ampicillin resistant culture plate without IPTG. pTRDTa1GmaR-H with active HHRz was used as positive control and pTRDTa1GmaR-Hm with inactive HHRzm was construed as a negative control.

More than three single colonies of each HHRz were inoculated in 1ml LB medium with ampicillin and cultivated in 37°C and 250 rpm until OD_600_ of culture medium reach 0.3. The cells in the logarithmic phase were induced by IPTG (final concentration: 1 mM) in 37°C and 150 rpm for 18 h. Then *Escherichia coli* cells were centrifuged in 4°C and 4000 rpm for 5 min and washed with PBS twice to abolish LB medium. Cell pellets were resuspended by PBS and analyzed by FCM (Green fluorescence, excitation at 480 nm, emission at 510 nm; Red fluorescence, excitation 561 nm, emission 610 nm). EGFP was fusion expressed with mCherry and used as internal reference. Only cells with normal green fluorescence would be analyzed by FCM.

### *In vivo* cleavage activity of selected HHRz variants in zebrafish (*Danio rerio*)

Eukaryotic vector construction of *trans*-cleaving HHRz targeted *mCherry* was described in Supplementary Figure S22. WT adult male and female zebrafish, Danio rerio, were maintained in 30 gal aquaria at 28.5°C on a 14:10 light–dark cycle. Fertilized embryos were obtained after natural spawning, washed, distributed into 20 × 100 mm culture plates and maintained at 28.5°C. AB strains were used in our studies. All zebrafish experiments were performed in accordance with the guidelines of the animal ethical committee of West China Hospital. All experimental protocols were proved by the Animal Ethical Committee, West China Hospital, Sichuan University.

Recombinant vectors were prepared by AxyPrep Endo-Free Miniprep kit (Axygen) and quantified by NanoDrop Microvolume Spectrophotometers and Fluorometer (Thermo Fisher). The plasmids were injected into the yolk at the one cell stage. Unless stated otherwise, a volume of 1 nl was injected into embryos with the concentration of 300 ng/μl of the plasmid. For morphological assessment, embryos were raised to 24–30 hpf and imaged, with one exception, *nacre* was raised to 50 hpf and imaged by microscope (ZEISS Axio Zoom.V16).

## RESULTS

### Strategy of direct intracellular selection of HHRz

To achieve intracellular selection of HHRzs, *E. coli* was chose as host cells to express mutant ribozymes library. Unlike the mammalian cell, each *E. coli* cell can only be transformed by a single plasmid, making the phenotype selection of ribozyme variants feasible. The given phenotype of host cells should be determined by the performance of HHRz that would cleave the reporter mRNA in the cell, thus reducing the expression of the corresponding gene to produce a negative signal. A positive phenotype signal is more desirable since the negative signals could be caused by various intracellular and extracellular factors. In this study, a 19-amino-acid toxin protein (IbsC) was applied as a reporter, which is highly hydrophobic and anchored in the inner membrane of *E. coli*. Overexpression of this protein can compromise the membrane's integrity and result in its depolarization (32–34). Extensive mutation can be tolerated by IbsC without loss of toxicity, and fusion of other proteins at its N terminus could be feasible without toxicity compromising. These characteristics qualify *ibsC* as a potential reporter gene for intracellular selection of gene-silencing tools.

To test the feasibility of application of *ibsC* for intracellular evolution of RNA-cleaving ribozyme, wild-type hammerhead ribozyme (WT-HHRz) sequence was inserted between ribosomal binding site (RBS) and ORF (open reading frame) of *ibsC* on vector to regulate the expression of toxin protein through *cis-*cleavage activity (Figure 1B, Supplementary Figures S2–S4). When the transcription of *ibsC* was induced by IPTG, the self-cleavage of active HHRz in mRNA sequences could cause the suppression of downstream toxin gene due to their loss of upstream translation factors, thus the host cells surviving on the resistant culture plate would offer a positive phenotype (Figure 1B).

Recombinant plasmids containing WT-HHRz were electrotransformed into host cells and cultured overnight on agar plates with ampicillin and IPTG. As shown in Figure 1C, only *E. coli* with WT-HHRz grew normally on the IPTG plate, while no any colony was observed on the culture plate of host cells containing inactive ribozyme (HHRzm with an A to G mutation at 14nt of the catalytic core (35)) or negative control (containing empty vector). The *in vitro* cleavage assays of HHRz, HHRzm, and no HHRz were consistent with the observations of the culture plate (Supplementary Figure S5), indicating that the expression of *i bsC* gene could be well regulated by RNA-cleaving ribozyme, and the IbsC protein is a promising reporter for the evolution of HHRz by providing positive selection signals.

### Intracellular selection of *trans*-acting HHRzs based on IbsC toxin

**Figure 2.**
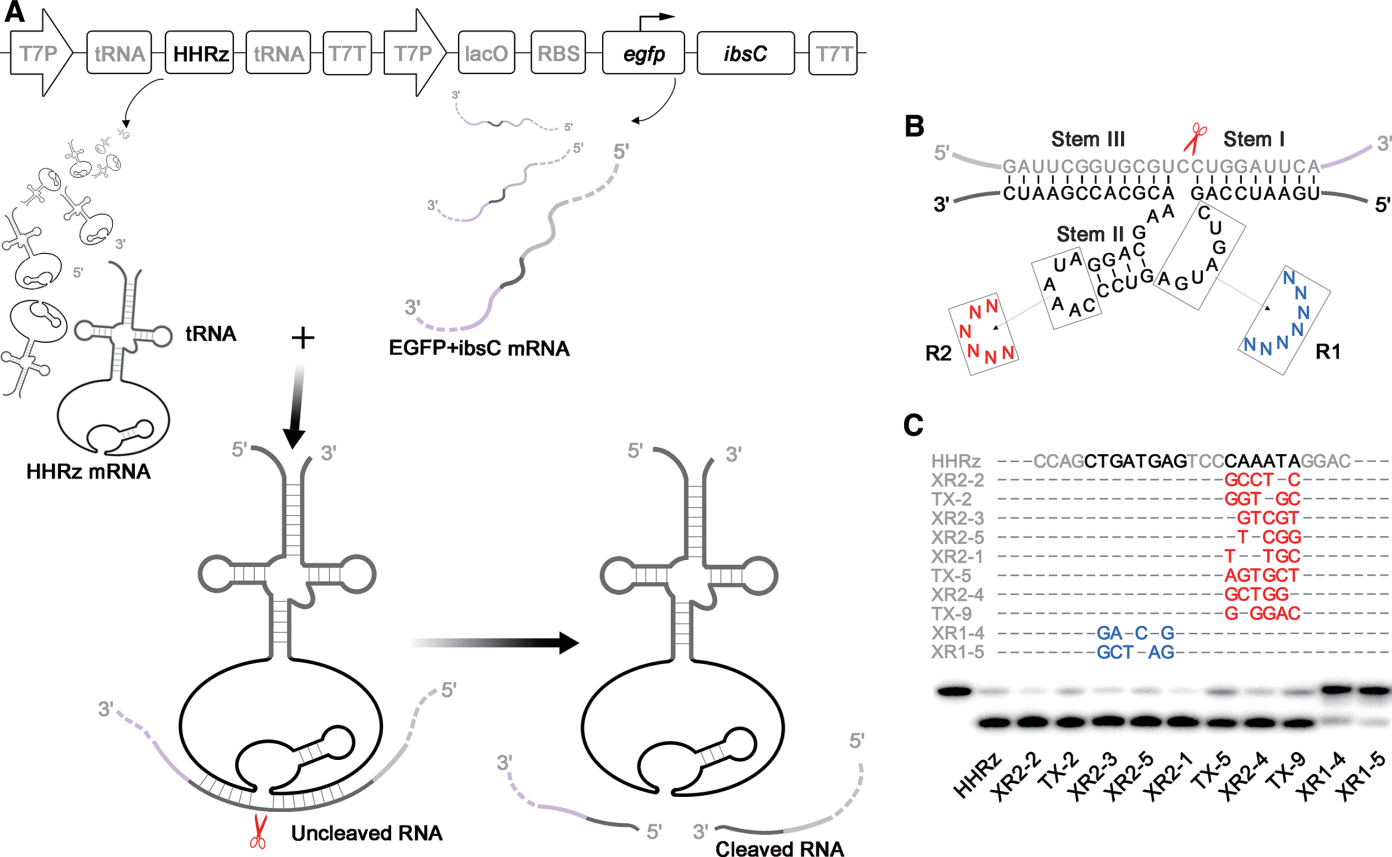
Intracellular selection of *trans*-acting HHRzs by IbsC toxin system. (**A**) The transcription of *trans*-acting HHRz are independent with that of *ibsC* mRNA. *Trans*-acting HHRz binds to substrate mRNA and cleaves the mRNA of *ibsC* to down-regulate the expression of toxin. (**B**) Structure of the randomized library used for intracellular selection. R1 contains 7-nt randomized region in catalytic core of WT-HHRz and R2 includes 6-nt randomized region in Loop II of WT-HHRz. (**C**) DNA sequence alignment of evolved HHRz variations and PAGE analysis of RNA substrate cleavage by HHRz mutants. Modified nucleotides in the catalytic core of the HHRz are highlighted in blue, and nucleotide mutations in Loop II are labeled in red. *Trans*-cleavage reactions were performed for 2 h at 37°C in 50 mM Tris–HCl (pH 8.4), 200 mM NaCl and 10 mM MgCl_2_. Lane 1, 5′-p32 labeled RNA substrate. Lane 2, cleavage of HHRz. Lane 3–12, cleavage of HHRz various variations.

Based on the IbsC toxin, systematic investigation of the intracellular selection was carried out for HHRz *trans*-cleavage. To achieve intermolecular action, HHRz and *ibsC* were expressed independently (Figure 2A). Unlike the spontaneous folding that induces the self-cleavage in *cis*-acting HHRz, the successful recognition and binding of mRNA substrate by the ribozyme is a prerequisite for *trans*-cleavage. Therefore, in our intracellular selection strategy, HHRz was integrated with anticodon loop of tRNA to prevent rapid digestion by RNases, and lacO operator in the upstream of HHRz was deleted to accelerate its transcription (Figure 2A), thus increasing the HHRz transcription, while minimizing the expression of *ibsC* gene in the cell relatively, resulting in a relative high ribozyme/substrate ratio. To further slow toxic protein expression, lacO operator was engineered between promoter and *ibsC* gene to work as a break for transcription, and *egfp* was fusion expressed with *ibsC* to extend the transcription and translation process of the reporter gene (Figure 2A). The cleaving site was located in an artificial linker sequence between *egfp* and *ibsC* which can be recognized by HHRz via Watson-Crick base-pairing (Supplementary Figure S10).

A mutant HHRz pool containing random domains in the catalytic core or loop II of ribozyme was constructed to cleave the targeted sequence, from which we intended to select HHRz variants that could achieve high *trans*-cleaving efficiency (Figure 2B). The hammerhead ribozyme with randomized sequence was inserted into selection vectors and recombinant plasmids were transformed into host cells (transformation efficiency>10^8^PFU), then incubated on the agar plate with IPTG to induce the expression of the toxin. From 10^8^ transformed bacteria, dozens of colonies survived on selection plate, and ten hammerhead ribozyme variants were finally harvested after excluding the replicated sequences, among which eight with mutant loop and the rest two with mutant catalytic core (Supplementary Figure S11). Based on the isotope-labeled experiments *in vitro*, all ten variants revealed *trans* RNA-cleaving catalytic activity in the reaction with 10 mM Mg^2+^, and HHRz variants bearing mutations in loop domain gave better results than HHRz variants with mutations in the catalytic core (Figure 2C, Supplementary Figure S12).

### Intracellular selection of *trans*-cleaving HHRz by dual fluorescent proteins

**Figure 3.**
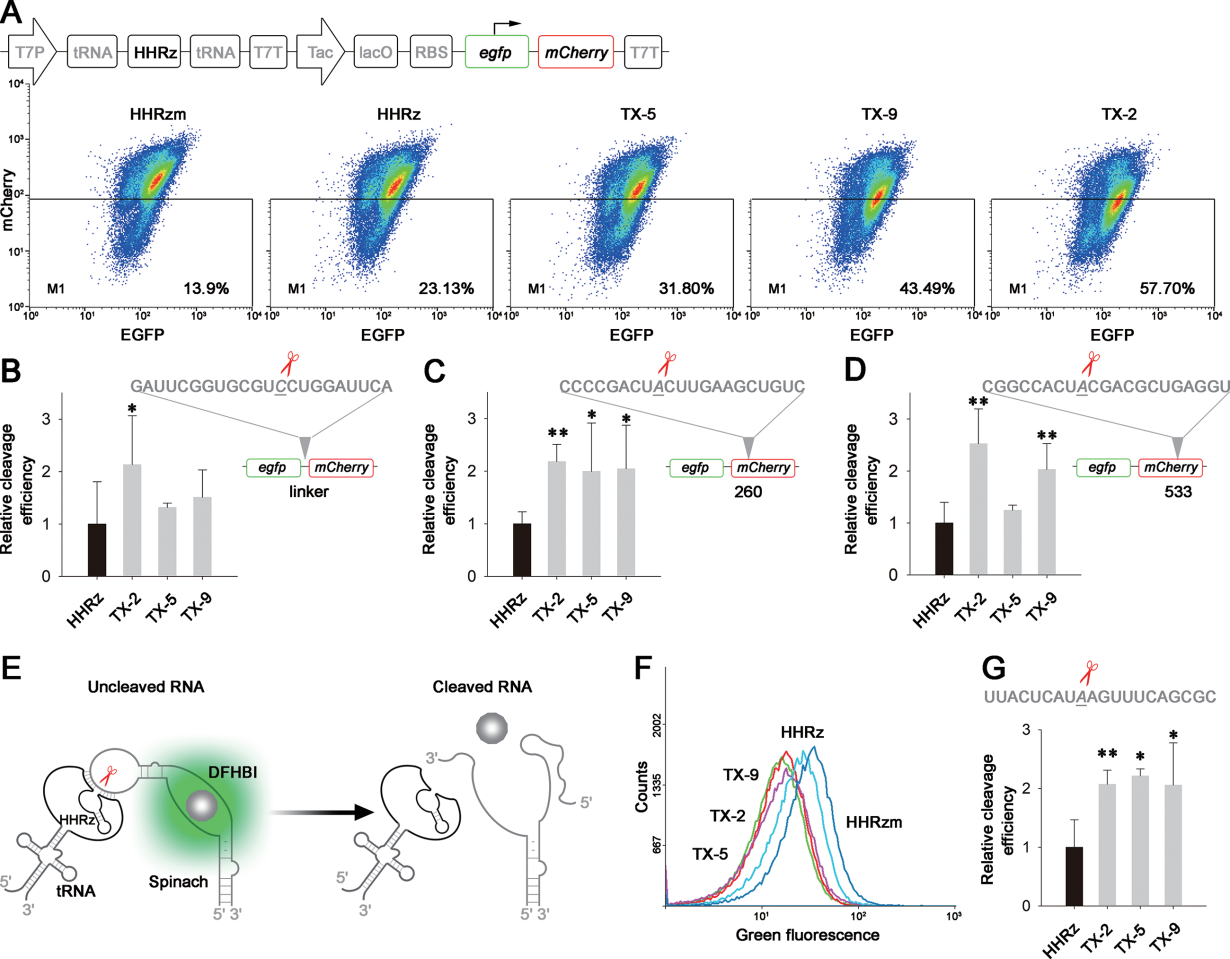
Intracellular definition of *trans*-cleaving HHRz by dual fluorescent proteins and RNA Spinach. (**A**) Intracellular characterization of *trans*-cleaving HHRz by dual fluorescence proteins system. *E. coli* containing HHRz was cultivated to OD0.3 and induced by IPTG for 18 h, following analysis by FCM. FCM dot plots present the fluorescence distribution of BL21(DE3) transformed with different HHRzm, HHRz and selected HHRz mutants. (**B–D**) Histogram show the relative cleavage efficiency at targeted linker, 260, 533 respectively. Relative cleavage efficiency = (R_Hm_– R_X_)/(R_Hm_-R_H_); R_Hm_ = mCherry/EGFP ratio of HHRzm, R_H_ = mCherry/EGFP ratio of HHRz, R_X_ = mCherry/EGFP ratio of HHRz variants; Relative cleavage efficiency of HHRz was identified as 1. (**E**) Definition of cleavage activity for HHRz variants in RNA level. Active HHRz could cleave the RNA of Spinach and reduce the bacterial fluorescence. (**F**) Intracellular characterization of *trans*-cleaving by Spinach. *E. coli* expressing HHRz was cultivated to OD0.3 and induced by IPTG for 6 h, then analyzed using FCM. (**G**) FCM histogram showing the green fluorescent shift of HHRz variants. Relative cleavage efficiency = (R_Hm_– R_X_)/(R_Hm_– R_H_); R_Hm_ = fluorescence of HHRzm, R_H_ = fluorescence of HHRz, R_X_ = fluorescence of HHRz variants; Relative cleavage efficiency of HHRz was identified as 1. Compared with HHRz, *P< 0.05; **P< 0.01.

Though we obtained ten new HHRz variants through *in vivo* selection, it is difficult to quantitatively evaluate the intracellular catalytic ability of those selected HHRzs according to their *in vitro* cleaving efficiency or the *ibsC* based cell-survival experiments. Therefore, a novel method was developed to assess the intracellular performance of HHRz variants, in which fluorescence proteins were introduced as the reporter. Due to the low background fluorescence, *mCherry* was applied to replace the *ibsC* for assessment of the intracellular cleavage efficiency of HHRz (Supplementary Figure S13). However, the inconsistent growth of *E. coli* would affect the fluorescent intensity of mCherry and lead to over- or under-estimate the cleavage efficiency of HHRz mutants. Thus, *egfp* was fused into N-terminus of *mCherry* to work as an internal reference to normalize the expression of the red fluorescent protein. HHRzs targeted the artificial linker between *mCherry* and *egfp* to knockdown the expression of *mCherry* through cleaving its mRNA, while the upstream *egfp* could be expressed normally (Figure 3A, Supplementary Figure S13). As a result, the cleavage efficiency of HHRz variants could be accurately assessed by comparing the relative intensity ratio between mCherry and EGFP with flow cytometry (FCM).

Based on the dual fluorescence proteins system, three mutants of HHRz exhibited improved catalytic activity (Figure 3A, Supplementary Figure S14 and S15), and a stronger up to ∼2-fold suppression of mCherry expression was achieved by the best one (TX-2) compared with WT-HHRz (Figure 3B). To evaluate the generality of selected HHRz variants, different cleaving sites (260nt and 533nt) on the coding sequence of *mCherry* was selected as the target of ribozymes (Figure 3C and D, Supplementary Figure S16), and the corresponding HHRzs were constructed by changing their binding arms to hybrid the new RNA domain. According to the analytic results of FCM, TX-2 maintained constant performance at different positions on the targeted mRNA, affording the best results in suppression of the reporter gene (Figure 3C and D, Supplementary Figure S17 and 18).

### Intracellular selection of *trans*-cleaving HHRz at RNA level

Both toxin and fluorescence proteins reporting system indicate that the *trans*-cleavage of ribozyme successfully decrease the expression of the target gene at translational levels. To verify the loss-function phenotype is due to the cleavage of mRNA by the ribozyme, a novel reporting system based on post-transcription levels was constructed (Figure 3E). Spinach is an RNA mimic of GFP which can bind with a small molecule (DFHBI) to become brightly fluorescent by laser irradiation, thus making itself an ideal tool for live-cell RNA imaging (36). Spinach was introduced into our system as a new reporter, whose loop domain was selected as the target of ribozymes (Figure 3E). The corresponding HHRzs were constructed to disrupt the binding of Spinach RNA to the fluorophore through cleavage (Supplementary Figure S19 and S20). As shown in Figure 3F and G, the cellular fluorescence was significantly decreased in presence of active ribozymes when compared to that of inactive HHRzm, affording the direct evidence of *trans*-cleaving target RNA by HHRzs in the intracellular environment. In addition, all three HHRz variants displayed 2-fold higher cleaving efficiency than WT-HHRz (Figure 3F and G, Supplementary Figure S21), which was consistent with their knockdown ability of a given gene at the translation level.

### HHRz-based gene knockdown in cancer cell and zebrafish

**Figure 4.**
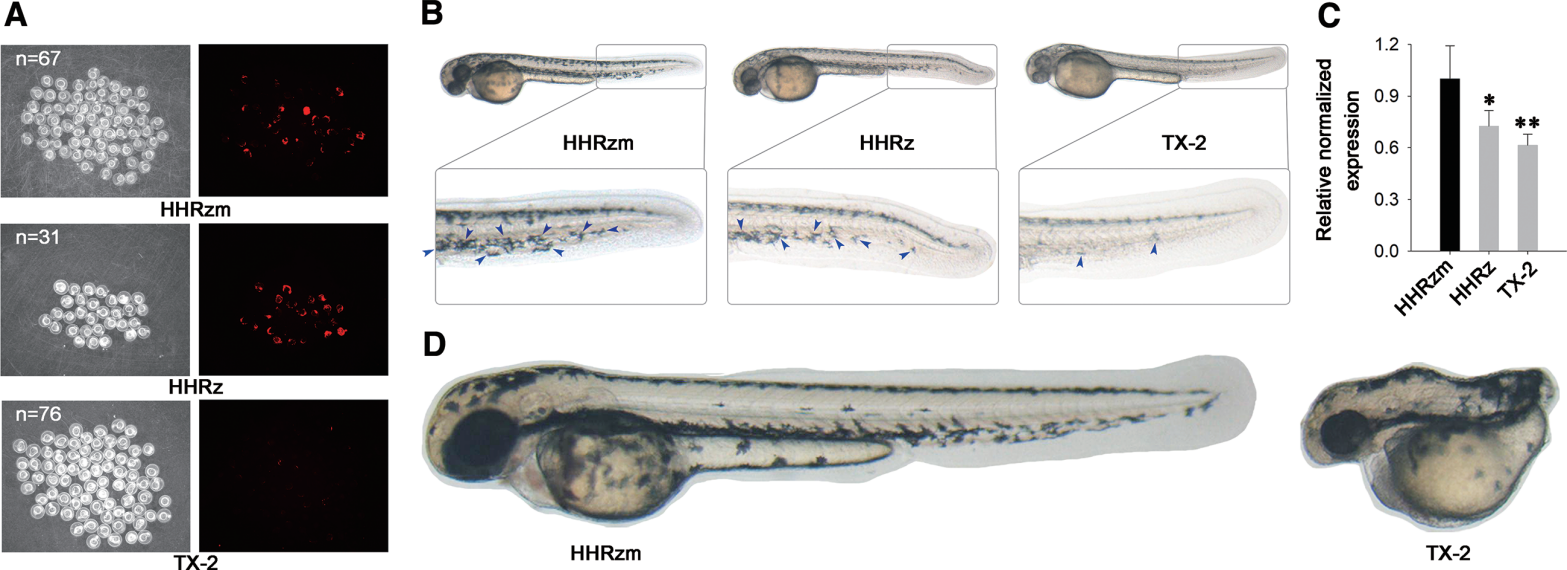
HHRz-based gene knockdown tools down-regulate gene expression in zebrafish. (**A**) mCherry gene inhibition by injected plasmid of HHRz. mCherry expression inhibition in 28 hpf embryos by injected TX-2 under FITC illumination. mCherry fluorescence in embryos injected HHRzm and HHRz was ubiquitous. (**B**) HHRz-based gene knockdown caused pigmentatione defects in zebrafish. Microscopy analysis of pigmentatione in HHRzm, HHRz and TX-2 plasmid injected 50 hpf embryos. The pigmentatione is highlighted by blue arrow. (**C**) Relative mRNA levels of *nacre* gene in HHRZm, HHRz and TX-2 plasmid injected 24 hpf embryos. Compared with internal reference gene, *P< 0.05; **P< 0.01. (**D**) HHRz-based gene knockdown led to no tail phenotype. Microscopy analysis of tail in HHRzm, HHRz and TX-2 plasmid injected 28 hpf embryos.

Given that all HHRz mutants were obtained from *E. coli* bacteria, their intracellular performances in eukaryotic cells is necessary to be investigated. Therefore, TX-2, TX-5 and TX-9 were applied to inactivate the expression of an exogenous non-essential *mCherry* gene by targeting its coding sequence in Hela cells. According to the analytical result of flow cytometry, at 48h post-transfection TX-2 in all HHRz variants provided the best suppression of the targeted gene (Supplementary Figure S23). The outstanding ability of TX-2 was further confirmed to suppress the same gene in fertilized zebrafish eggs through injection of plasmid DNA (Figure 4A, Supplementary Figure S25). The results revealed the potential application of TX-2 in gene silencing. Consequently, it was applied in the down-regulation of endogenous genes (*BCL2* and *GAPDH*) of the human cancer cell, showing that TX-2 knocked down expression of the target genes much more effectively than WT-HHRz in both mRNA and protein level (Supplementary Figure S24). In addition, an experiment to knock down the expression of a given gene of zebrafish was carried out to evaluate the efficacy of our new ribozyme tools. The *nacre* gene of zebrafish codes a transcription factor, Mitf1, is required by the cells to develop to the melanophore precursor. The functional loss of the *nacre* gene leads to the body pigmentation modification of zebrafish. Ribozymes were designed to target the coding sequence of *nacre* and injected into zebrafish embryos. As shown in Figure 4B, the pigmentation of zebrafish, especially in dorsal midline of the tail, was effectively suppressed by ribozyme tools (Supplementary Figure S26). In comparison with WT-HHRz, TX-2 variant achieved an enhanced suppression of *nacre* gene expression, which had been confirmed by mRNA quantification (Figure 4C). We then chose the coding sequence of no tail (*ntl*) gene as the target to verify further the knockdown ability of gene expression by TX-2 ribozyme. At 28 hours post fertilization, 27% embryos injected with *ntl*-targeted TX-2 ribozyme displayed typical no tail phenotype (Figure 4D, Supplementary Figure S27), which is indistinguishable from those caused by a null mutation (37). These loss-of-function phenotypes demonstrate that the TX-2 is an efficient molecular tool to inactivate the expression of the target gene through knockdown its mRNA specifically.

### Structure and Kinetic analysis of selected mutants

**Figure 5.**
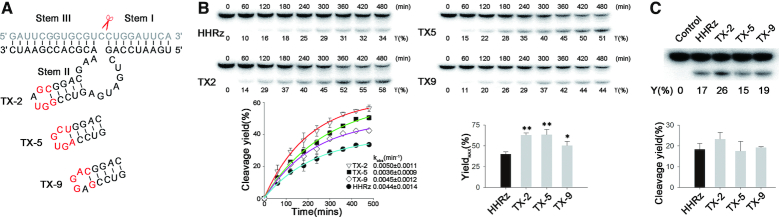
Predicted structure and Kinetic analysis of selected mutants. (**A**) Predicted structure of TX-2 by *m*-fold software. Modified nucleotides in Loop II are labeled as red color. (**B**) PAGE analysis of RNA substrate cleavage by HHRz mutants under single-turnover conditions (about 1 μM HHRz combined with 10 nM substrate RNA) at 37°C in 50 mM Tris–HCl (pH 7.4), 200 mM NaCl and 0.5 mM MgCl_2_. Representative results are shown for a 6-h time course; the observed rate constant was fit by using non-linear regression. Maximum cleavage yield (CYmax) of HHRz, TX-2, TX-5 and TX-9 under single-turnover conditions. (**C**) PAGE analysis of RNA substrate cleavage by HHRz mutants under multi-turnover conditions (about 10 nM RNA of HHRz mutants with a 10-fold excess of substrate) at 37°C for 6 h in 200 mM NaCl, 50 mM Tris–HCl (pH 7.4) and 0.5 mM MgCl_2_. These experiments were performed at least in triplicate and data shown are representative of three independent experiments.

It is remarkable that hammerhead ribozyme could be selected in the cell to improve their performance in the *trans*-acting cleavage. Though we obtained HHRz variants with a mutant catalytic core (XR1-4 and XR1-5, Figure 2C), all three HHRzs (TX-2, TX-5 and TX-9, Figure 5A) that displayed the enhanced gene-silencing capability in the cell retains the catalytic domain of WT-HHRz, which is highly conserved as a result of natural evolution. Based on *in vitro* isotope-labeled experiments, three new mutant HHRz variants afforded almost same *trans*-cleavage efficiency at high Mg^2+^ concentrations (data not shown). While, at the low Mg^2+^ concentrations, TX-2 exhibited the highest apparent first order rate constant (*k*_obs_) (Figure 5B), and all mutant HHRz afforded higher maximum cleavage yield (CY_max_) compared with WT-HHRz under single-turnover conditions (Figure 5B). It is well known that the activity of WT-HHRz strongly depended on the loop–loop interaction, particularly at low Mg^2+^ concentrations (16). Therefore, this result indicates that those three hammerhead ribozymes with new loop domains have been evolved to stabilize their active conformation without extra tertiary interacting elements. Meanwhile, under multiple turnover experiments condition (10:1 RNA substrate /Ribozyme), TX-2 afforded the highest cleaving efficiency as well (lane 3, Figure 5C), revealing its comprehensive improvement of catalysis (substrate recognition, cleavage and turnover) as a *trans*-acting ribozyme through intracellular selection.

## DISCUSSION

Without changing the genetic code, RNA-targeting therapeutics directly prevent the disease-propagating protein or enzyme from production at the mRNA level, which could avoid the risks and concerns associated with DNA-based gene therapies. In these applications, hammerhead ribozyme offers unique features. First, they are smaller than most other gene-regulation systems, typically <50 base pairs. Therefore, they can be synthesized and chemically modified easily or included in gene therapy vectors such as AAV with limited space for additional genes. Second, the *trans*-acting ribozymes bind to the mRNA substrates through two recognition arms that are complementary to the target sequence, and the cleaving site in the mRNA substrate need to fulfill ‘NUX**↓**’ rule. They are therefore highly specific, with few off-target effects.

Here, we describe a direct evolution strategy for converting the self-cleaving HHRz into *trans*-acting gene knockdown tools in the cell. Based on IbsC toxin protein, HHRz variants that provide positive phenotype signals at the cellular level have been selected. This strategy not only reduces the false positive result significantly but also saves time and labor of selection, offering a simple and effective way to evolve *trans*-cleaving ribozyme. To further evaluate the ability of those ribozymes to inactivate the expression of a specific gene in the cell, a dual fluorescence proteins system has also been established. The efficiency of each HHRzs variant was accurately quantified with flow cytometry analysis, showing that three HHRz variants offerred enhanced intracellular activity compared with WT-HHRz. The best one, TX-2 revealed the better and consistent gene knockdown ability at different positions on diverse targeted mRNA either in prokaryotic or eukaryotic cells, indicating that the HHRz mutant no longer relies on the loop-loop interaction to fold into the right structure to achieve high *trans*-cleaving activity. Using TX-2 as a novel molecular tool, we effectively generated phenotypes of mutations of the no tail and *nacre* genes in zebrafish. These experiments demonstrate the efficiency of intracellular selection method of *trans*-acting ribozyme. Although the knock-down efficiency of TX-2 was not so effective as RNA interference or CRISPR interference, it is still a useful *trans*-acting tool for research and therapeutic purposes due to its improved *trans*-cleavage activity.

## Supplementary Material

Supplementary Data
