# Supplementary material for: Intracellular selection of trans-cleaving hammerhead ribozymes
Source: Nucleic Acids Res. 2019 Jan 16;47(5):2514–22. doi: 10.1093/nar/gkz018 (PMC6412130; doi:10.1093/nar/gkz018)
Supplement: Supplementary Data [file gkz018_supplemental_files.pdf]

Supplementary Information

Intracellular Selection of *trans*-cleaving Hammerhead Ribozymes

Xin Huang,[1†] Yongyun Zhao,[2†] Qinlin Pu,[1] Getong Liu,[1] Yan Peng,[1] Fei Wang,[1] Gangyi Chen,[1] Meiling Sun,[1] Feng Du,[1] Juan Dong,[1] Xin Cui,[1] Zhuo Tang[1]* and Xianming Mo[2]*

[1]Natural Products Research Center, Chengdu Institution of Biology, Chinese Academy of Science, Chengdu 610041, P. R. China. E-mail: tangzhuo@cib.ac.cn; Fax: +86 28 8524 3250; Tel: +86 28 8524 3250

[2]Laboratory of Stem Cell Biology, State Key Laboratory of Biotherapy, West China Hospital, Sichuan University, Chengdu 610041, China. E-mail: xmingmo@scu.edu.cn

Fax: +86 28 8516 4047; Tel: +86 28 8516 4047

[†] The first two authors contributed equally to this work

Table of Contents

Supplementary Information 1
Intracellular Selection of *trans*-cleaving Hammerhead Ribozymes 1
S1 Supplementary text 3
S1.1 Structure of hairpin RBz, HDV RBz and HHRz 3
S1.2 Feasibility of intracellular selection strategy by using toxin protein 4
S1.2.1 Vector construction of *cis*-cleaving HHRz 4
S1.2.2 Intracellular comparison of initiation efficiency of T7 and Tac promoter 6
S1.2.3 Intracellular cleavage assays of *cis*-cleaving HHRz 8
S1.2.4 *In vitro* isotope-labeled assays of *cis*-cleaving HHRz 9
S1.3 *trans*-cleaving HHRz selection based on IbsC 10
S1.3.1 Vector construction of *trans*-cleaving HHRz 10
S1.3.2 Prevention of intracellular rapid degradation of HHRz mRNA by tRNA scaffold 11
S1.3.3 Relative higher expression of HHRz 13
S1.3.4 Intracellular selection of *trans*-cleaving HHRz 14
S1.4 *In vitro* cleavage expriments of WT-HHRz and mutants 16
S1.5 Definition of intracellular cleavage efficiency of selected HHRz mutants by double fluorescent protein system 17
S1.4.1 Vector construction of *trans*-cleaving HHRz targeted mCherry 17
S1.4.2 Expression of HHRz and fluorescent protein by different promoters 18
S1.4.3 Intracellular definition of *trans*-cleaving HHRz targeted linker sequence 19
S1.4.4 Selection of target sites on mCherry mRNA 21
S1.4.5 Intracellular definition of *trans*-cleaving HHRz targeted 260 and 533 of mCherry 22
S1.6 Definition of intracellular cleavage efficiency of selected HHRz mutants at RNA level 24
S1.5.1 Vector construction of *trans*-cleaving HHRz targeted Spinach 24
S1.5.2 Intracellular definition of *trans*-cleaving HHRz targeted various Spinach and tRNA chimeras 26
S1.7 Intracellular cleavage efficiency of HHRz variants in human cancer cells 28
S1.6.1 Eukaryotic vector construction of *trans*-cleaving HHRz 28
S1.6.2 Intracellular cleavage efficiency of HHRz variants target mCherry in *Hela* cells 29
S1.6.3 Intracellular cleavage efficiency of HHRz variants target endogenous genes in cancer cells 30
S1.8 Intracellular cleavage activity of HHRz variants in *zebrafish* 31
S1.7.1 Intracellular cleavage efficiency of HHRz variants target *mCherry* in *zebrafish* 31
S1.7.2 Intracellular cleavage efficiency of HHRz variants target *nacre* in *zebrafish* 32
S1.7.2 Intracellular cleavage efficiency of HHRz variants target *ntl* in *zebrafish* 33
S2 Material and methods 34
S2.01 Library design and Synthesis of random single-strand DNA sequence 34
S2.02 Intracellular evolution of *trans*-cleaving HHRz by toxin protein 34
S2.03 Definition of *trans*-acting mutants based on dual fluorescent proteins using FCM 34
S2.04 Definition of *trans*-acting mutants variants via Spinach 34
S2.05 *In vitro* transcription of RNA 35
S2.06 p^{32} label of RNA substrate 35
S2.07 *In vitro* cleavage assay 35
S2.08 Kinetic analysis of selected clones 35
S2.09 Multi-turnover Assay of Selected variants 36
S2.10 Intracellular cleavage activity of selected HHRz variants target mCherry in Hela cells 36
S2.11 Intracellular cleavage activity of selected HHRz variants target other genes in cancer cells 36
S2.12 RT-PCR of cancer genes 36
S2.13 Western blotting (WB) of cancer genes 37
S2.14 Intracellular cleavage activity of selected HHRz variants in *zebrafish* 37
S2.15 RT-PCR of *zebrafish* genes 37
S3.Supplementary Table S1. Primers of plasmid construction and *in vitro* transcription 38
S4.Supplementary Table S2. Plasmids used in the work 40
References 41

S1 Supplementary text

S1.1 Structure of hairpin RBz, HDV RBz and HHRz

Small catalytically active nucleic acids range from 30 to ~150 nucleotides in length which catalyze intramolecular splicing or cleavage reactions. The hammerhead ribozyme is the smallest, most intensively studied and applied ribozyme to date. The development of HHRz mutants that *trans*-cleave RNA substrate was a major advancement to improve the use of ribozyme tools for practical applications. The hairpin ribozyme is the second smallest catalytic RNA molecule. A minimal catalytic one which consist two domains with a functional length of 50 nucleotides has been identified to catalyze cleavage reactions in *trans*. The minimal natural HDV ribozyme is about 88 nucleotides long. *Trans*-acting derivatives of HDV ribozyme have been developed (Supplementary Figure 1). These RNA trans-cleaving enzymes which can cleave target RNAs could be promising new tools for functional investigations and therapeutic applications. The sequence requirements for *trans*-cleaving HHRz, Hairpin ribozyme and HDV ribozyme were showed as follows.

HHRz recognize substrate by two binding arms and cleave adjacent to the sequence NUX↓ (N is any base and X is A, C or U). To design a therapeutic tool of HHRz, it is important to selects a region in the target RNA containing NUX first. The target sequences were located outside the tightly matched stem in the second structure of mRNA of target gene. Second, two arms of 8~9 nucleotides in length that flank the 24 nucleotide catalytic core forming the catalytic hammerhead between them are designed based on the target sequence surrounding the third nucleotide (X) of the target triplet. It is better to maintain the GC ratio of binding arm ranged from 40~60% (Supplementary Figure 1).

The catalytic core of Hairpin ribozyme is larger (40 nucleotides). Hairpin ribozyme cleaves RNA substrate in the position of NGUC and the ribozyme targeting domains require more specificity. Hairpins recognize the sequence NNNYNGUCNNNNNN, where N is any nucleotide and Y is a pyrimidine. The underlined target bases (NGUC) are not base-paired with the ribozyme. Two arms of 4 and 6 nucleotides in length respectively that flank the 40 nucleotide catalytic core between them are designed based on the target sequence surrounding the NGUC sequence (Supplementary Figure 1).

HDV ribozyme cleaves the RNA substrate at the base of the P1 helix, typically at a guanosine residue. HDV ribozyme recognize the sequence (n)GNNNNNN(n)NNNNNNN, where (n) is any nucleotides for connecting and N is any nucleotide for ribozyme binding. Two arms of 7 and 7 nucleotides in length respectively that flank the a much longer catalytic core (71 nucleotide) in the downstream of G are designed based on the target sequences, especially the cleaved G on substrate should be bound with a pyrimidine (Supplementary Figure 1).

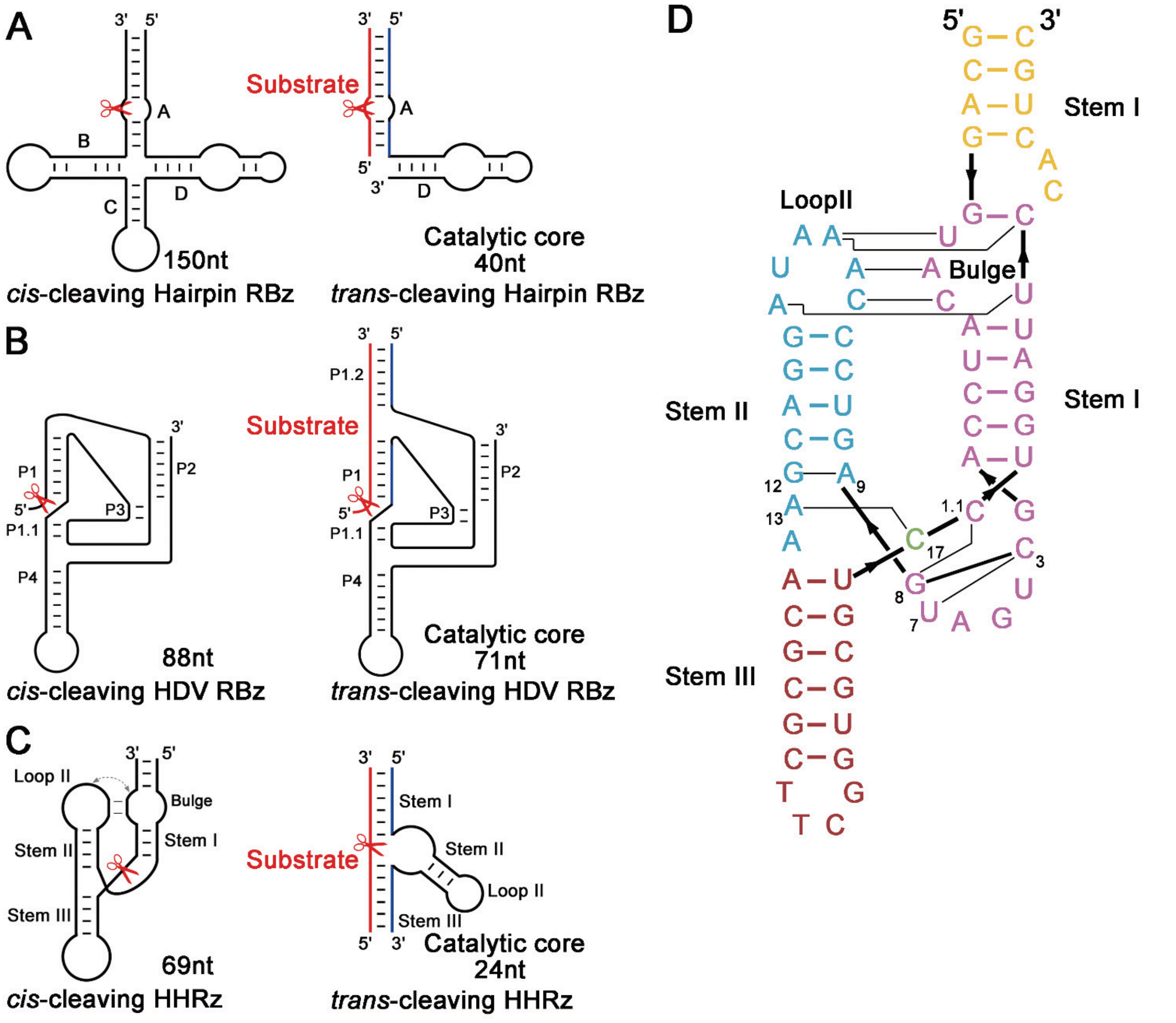

Supplementary Figure S1. Structure of hairpin RBz, HDV RBz and HHRz: A, structure of *cis*- and *trans*-cleaving hairpin RBz; B, structure of *cis*- and *trans*-cleaving HDV RBz; C, structure of *cis*- and *trans*-cleaving HHRz; D. Topological structure of HHRz. Cleavage sites were identified by red scissors; Substrate RNA was labeled as red; Binding domain of *trans*-cleaving ribozyme was labeled as blue; Catalytic core of *trans*-cleaving ribozyme was labeled as black.

S1.2 Feasibility of intracellular selection strategy by using toxin protein

S1.2.1 Vector construction of *cis*-cleaving HHRz

The *cis*-cleaving HHRz expression cassettes and vectors are described in Supplementary Figure S2. Firstly, to insert full length *ibsC* sequence into vector pET32a, two primers ibsC-5H and ibsC-3X (which is partially complementary with each other) (Supplementary Table S1) were amplified into dsDNA by polymerase chain reaction (PCR) to form full length DNA of 60 nucleotides (5'-ATGATGCGACTTGTCATCATACTGATTGTACTGTTACTCATAAGTTTCAGCGCTTATTAA-3'). 5' end and 3' end of dsDNA was flanked by restriction endonuclease site *Hin*dIII and *Xho*I (Supplementary Figure S2, step 1).The overexpression of IbsC toxin is lethal to *E. coli* [1].

In our strategy, the selection of HHRz mutants depends on the down regulation of IbsC. *E. coli* with inactive HHRz variants could not survive on the plate due to overexpression of ibsC. If its expression is too strong, the cleaving capacity of active HHRz mutant could not decrease the lethal effect of *ibsC*. Even cells with active HHRz could not grow in the selection process, which would make our intracellular selection method impossible. So the toxin need to be transcribed by promoter with appropriate initiative efficiency, and the expression of IbsC should be controlled by repressor protein LacI to facilitate the genetic manipulation of plasmid.

Two strong promoters, T7 promoter and Tac promoter, both were tested in this work to initiate the overexpression of toxin. pET32a is commercial devised plasmid with T7 promoter. Four primers EI5, Tac2, TaR2c and Tac3R (Supplementary Table S1) were amplified by PCR to form dsDNA of Tac promoter fragment. This fragment was inserted into pET32a to replace the T7 promoter at *SgrA*I and *Xba*I site (Supplementary Figure S2, step 4).

In original pET32a, there are too many tags for protein expression. To reduce the effect of unconcerned sequences on the folding and cleaving of HHRz, the sequence between initiation codon and HHRz need to be decreased to a larger extent. Two primers, RBS5 and RBS3 (Supplementary Table S1), were amplified into dsDNA and applied to substitute these irrelevant sequences at *Xba*I and *Bam*HI site (Supplementary Figure S2, step 2).

Between initiation codon and gene of ibsC, there are 25 nucleotides which would lead to the reading-frame shift of *ibsC*. So two primer BH-5 and BH-3 (Supplementary Table S1) were applied to replace the sequence (GGATCCGAGCTCCGTCGACAAGCTT) into 27 nucleotides DNA sequence (GGATCCGAGCTCCGTCGTTACAAGCTT) at *Bam*HI and *Hin*dIII site (Supplementary Figure S2, step 3). Finally, we constructed two plasmids, termed as pT7Oi, and pTaOi, which could be used to test the initiation efficiency of ibsC.

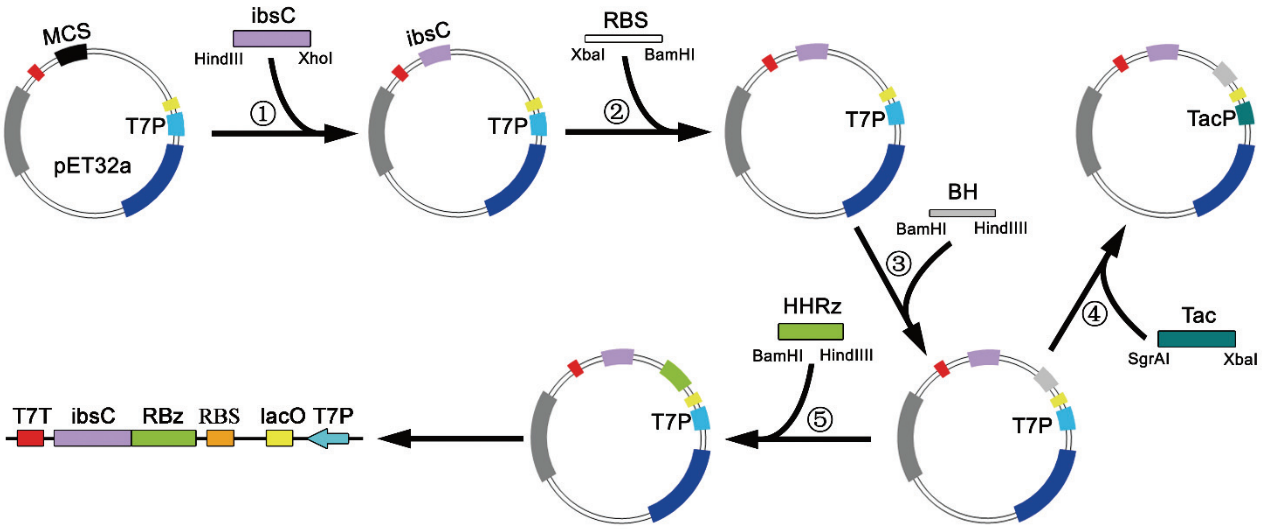

Supplementary Figure S2. Vector construction of *cis*-cleaving HHRz. Step 1, *ibsC* DNA fragment was amplified and inserted into pET32a in *Hin*dIII and *Xho*I sites. Step 2, dsDNA sequence containing RBS was cloned into vectors in *Xba*I and *Bam*HI site to remove the redundant sequences between promoter and initiation codon of *ibsC*. Step 3, BH DNA PCR products was constructed into plasmid in *Bam*HI and *Hin*dIII site to delete the frame shift after initiation codon of *ibsC*. Step 4, Tac DNA fragment was inserted into recombinant plasmid in *SgrA*I and *Xba*I site to construct a vector which initiates the expression of *ibsC* by Tac promoter. Step 5, HHRz were inserted into pT7Oi in *Bam*HI and *Hin*dIII site to generate the vector containing HHRz. MCS, multiple cloning site; T7P, T7 promoter; RBS, a DNA fragment with RBS to delete expression tags of pET32a; BH, a DNA fragment to prevent frame shift of ibsC; TacP, Tac prompter; RBz, ribozyme.

S1.2.2 Intracellular comparison of initiation efficiency of T7 and Tac promoter

Regardless of the promoters, cells would grow on the resistant plate without IPTG induction (Supplementary Figure S3A and S4A). With the induction of IPTG, overexpression of IbsC initiated by T7 promoter was deleterious to host strain cells; no one clone would survive on the culture plate (Supplementary Figure S3B). However, even in the induction of IPTG, Tac promoter could not initiate overexpression effectively. Although the clone number is less than that of *E. coli* without IPTG induction, there were still some survival *E. coli* cells on the resistant plate (Supplementary Figure S4B). Therefore in following experiments, pT7Oi containing T7 promoter was applied to initiate the overespression of IbsC. These observations meant that the expression of toxin protein regulated by HHRz might be feasible.

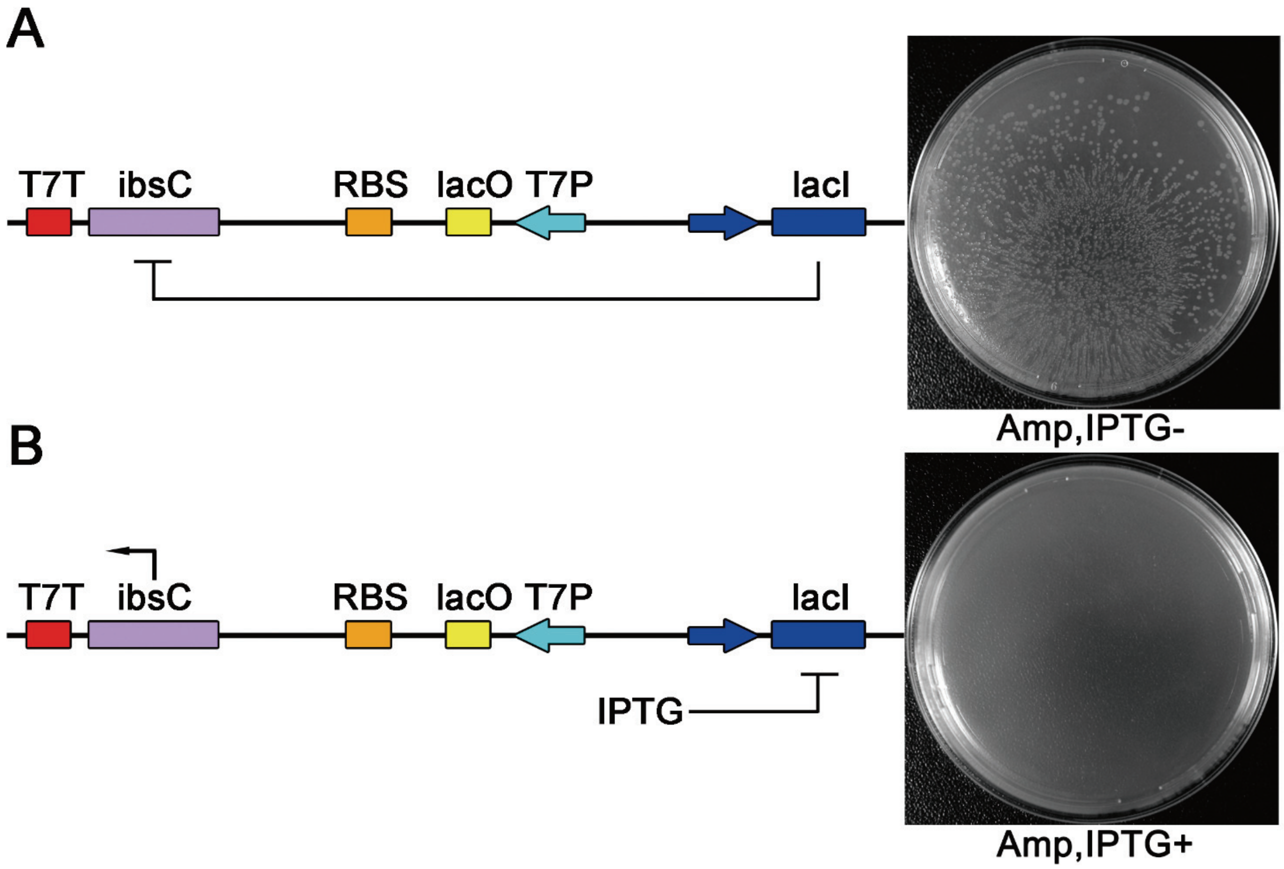

Supplementary Figure S3. Intracellular regulate expression of *ibsC* initiated by T7 promoter. BL21(DE3) cells were transformed by pT7Oi and cultivated at 37 ºC and 250 rpm. After incubation for 1h, 50μl transformed cells were coated on the resistant plate. Without the presence of IPTG, the expression of *ibsC* was suppressed by a repressor LacI and *E. coli* would grow on the plate (A). In the induction of IPTG, the expression of *ibsC* was initiated by T7 promoter and no cells survived on the plate (B). Amp, ampicillin; IPTG, isopropyl-β-d-thiogalactoside; T7T, T7 terminator.

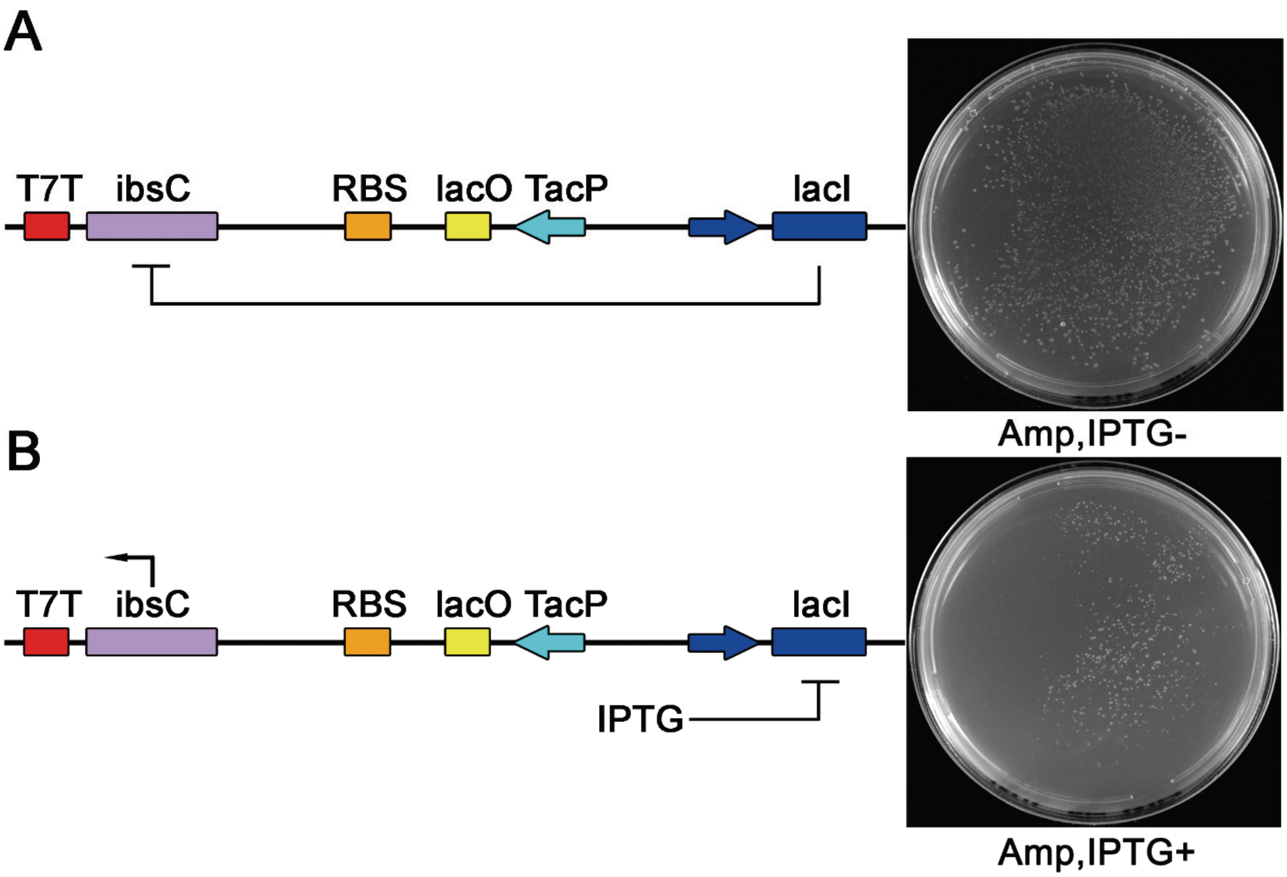

Supplementary Figure 4. Intracellular regulate expression of *ibsC* initiated by Tac promoter. BL21(DE3) cells were transformed by pTaOi and cultivated at 37 ºC and 250 rpm. After incubation for 1h, 50μl transformed cells were coated on the resistant plate. With (A) or without (B) IPTG, the expression of *ibsC* couldn't be regulated by IPTG and the cells would grow on the plate.

S1.2.3 Intracellular cleavage assays of *cis*-cleaving HHRz

Now that the overexpression of IbsC initiated by T7 promoter have been proved to be lethal to host cells in the induction of IPTG. HHRz was constructed into plasmid pT7Oi to test whether HHRz cleave the mRNA of IbsC toxin in cell. The HHRz could regulate the expression of IbsC toxin and make the fate of *E. coli* on resistant culture plate. *ibsC* was located in the downstream of HHRz and fusion expressed with HHRz. Inactive HHRzm could lead to the sequential expression of *ibsC* and caused death of host strain cells (Supplementary Figure S5A). Self-cleaved active HHRz would suppress the expression of downstream *ibsC* due to the deletions of gene regulatory elements of *ibsC* and *E. coli* with active HHRz could survive on the resistant culture plate theoretically (Supplementary Figure S5A). To exclude the possibility that inactive HHRz form secondary structure and subsequentially affect the expression of *ibsC*, the active core of HHRz was modified and catalytic activity was deleted to work as a negative control, termed as HHRzm (Figure 3A). Two primers HHRza5 and HHRza3 (Supplementary Table S1) were amplified by PCR into dsDNA. Simultaneously, an inactive HHRz termed HHRzm was inserted into the plasmid to work as negative control. The resulting products flanked by *Bam*HI and *Hin*dIII sites was digested and ligated in a 20 μl ligation reaction using T4 DNA ligase with a similarly digested pT7Oi expression cassette. Recombinant vectors were confirmed by DNA sequencing and transformed into Bl21(DE3) host strain cells.

As shown in Supplementary Figure S5B, only *E. coli* containing active HHRz survived on resistant culture plate with the induction of IPTG. *E. coli* with HHRzm (negative control) or without HHRz (blank control) both couldn’t grow on the plate. But all *E. coli* could survive on the resistant culture plate without IPTG, which illustrated that the bacterial survival might owe to the cleaved activity of HHRz but not the inconformity of growing status of *E. coli*.

S1.2.4 *In vitro* isotope-labeled assays of *cis*-cleaving HHRz

To further confirm the observations of culture plate, the cleaved activity of HHRz was evaluated by *in vitro* isotope-labeled experiments. The substrate DNA was prepared by primer RzaS5m4 containing T7 promoter sequence and RzaS3a by using fusion PCR. And ribozymes DNA were amplified by primer RzaF1 containing T7 promoter sequence and Rza-sR1 or Rzam-sR1 to generate HHRz and HHRzm. The RNAs were transcribed by T7 polymerase and purified by PAGE. As shown in Supplementary Figure S5, the results of in vitro cleavage assay were consistent with that of intracellular test. Only HHRz can cleave mRNA into separate small fragments (Supplementary Figure S5C), whereas HHRzm or no HHRz didn't generate cleaved products.

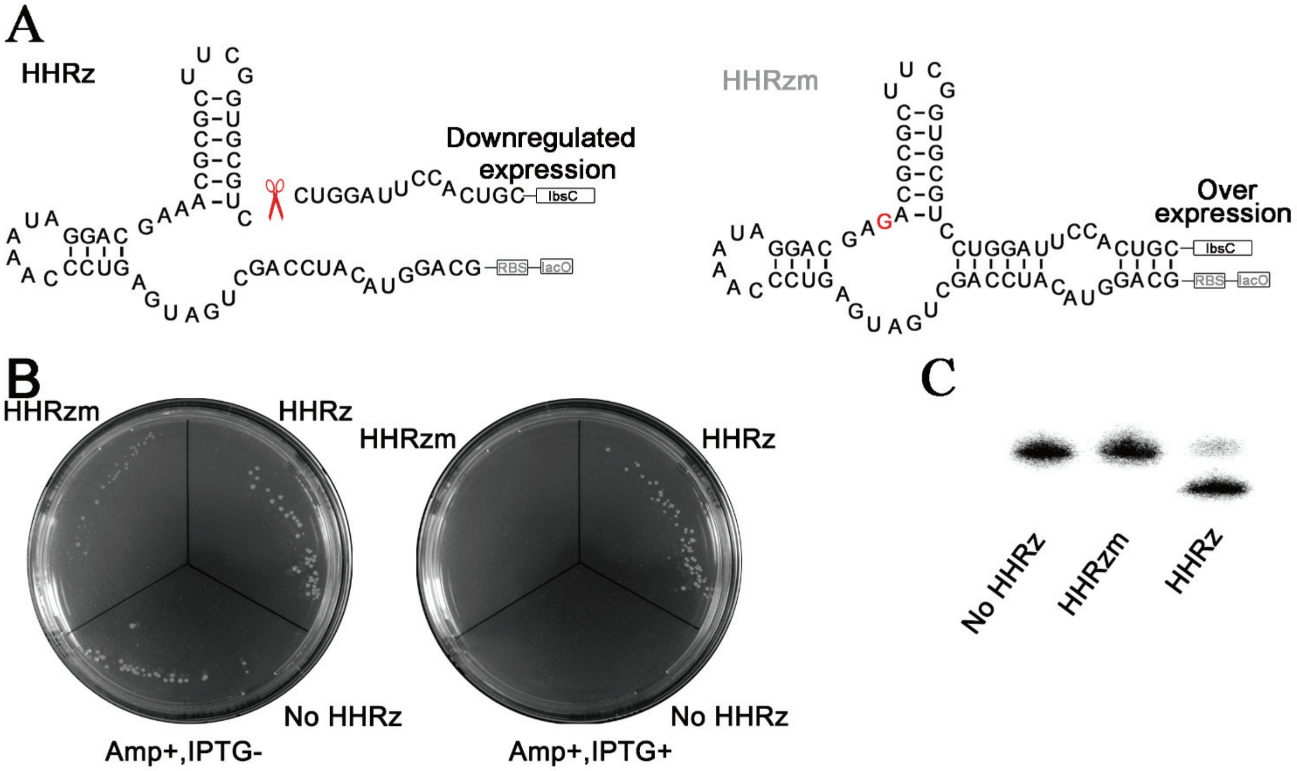

Supplementary Figure S5. Feasibility of intracellular selection strategy based on the expression of toxin IbsC regulated by *cis*-acting HHRz. A, mechanism of *cis*-acting HHRz down-regulate the expression of *ibsC*. active HHRz could cleave itself, and *ibsC* wouldn't be expressed due to the lack of promoter, RBS and ATG which located in the upstream of HHRz sequence; inactive HHRz (HHRzm) couldn't cleave itself and the downstream of *ibsC* would be expressed to the death of cells. B, intracellular defining for capacity of HHRz. BL21(DE3) competent cells were transformed by vectors with HHRz or without HHRz.; After 1h cultivation, transformed cells were coated on the ampicillin plate with IPTG and without IPTG for overnight. C, *in vitro* cleavage of HHRz. Trans cleavage reactions were performed for 2h in 10mM $MgCl_2$ at pH 8.4 and 37 ºC as described in the Materials and Methods.

Taking the results above together, intracellular selection method based on IbsC might render for the evolution of HHRz in cell. Therefore, a selection method based on ibsC toxin protein was directly used to generate trans-cleaving RBzs in cell. To generate HHRz that cleave RNA in physiological conditions, no extra Mg^{2+} was added into the *E. coli* selection process.

S1.3 *trans*-cleaving HHRz selection based on IbsC

S1.3.1 Vector construction of *trans*-cleaving HHRz

To evolve *trans*-acting HHRz mutants, the transcription of *trans*-acting HHRz is necessarily independent of expression of *ibsC*. As shown in Supplementary Figure S6 step 4 and 5, five primers T7TFS, T7TFL.1, iT7-1b, iT7-2bR and iT7-3aR (Supplementary Table S1) were amplified to dsDNA containing new regulatory elements (consisting of RBS, lacO operator and T7 promoter of ibsC, T7 terminator of HHRz) to initiate the expression of *ibsC* and terminate the transcription of HHRz, respectively.

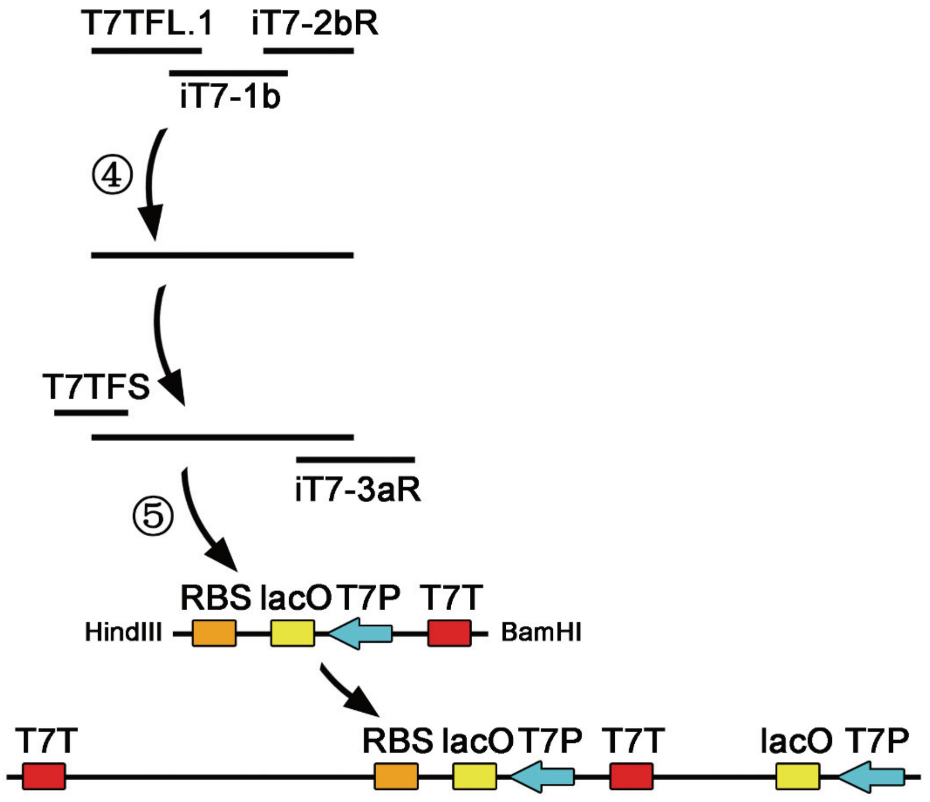

Supplementary Figure S6. Construction of plasmid for *trans*-acting HHRz selection. Independent elements of gene regulation for *ibsC* expression were prepared by two round of PCR; Step 4, T7TFL.1, iT7-1b and iT7-2bR were used in the first round PCR; Step 5, T7TFS, iT7-3aR and PCR products of first round were used to yield full length DNA products that then cloned into plasmid at *Hin*dIII and *Bam*HI site.

S1.3.2 Prevention of intracellular rapid degradation of HHRz mRNA by tRNA scaffold

E. coli is prokaryote with property that transcription, translation and degradation of mRNA is carried out simultaneously. Previous studies have showed that tRNA scaffold could prevent RBz from rapid digestion but not disturb the function of tRNA or RBz [2]. So HHRz were integrated with anti-codon loop of tRNA to enhance resistance of HHRz to intracellular RNase. 12/10z and 12/10zR were amplified to generate 5' part of tRNA sequence (Supplementary Figure S7, step 1). ai-H or ai-Hm, ai-H2 or ai-H2m and R-SR54 were amplified to produce 3' part of tRNA and HHRz sequence (Supplementary Figure S7, step 2). Intact tRNA-HHRz chimera is formed by PCR using three DNA fragments, 3' part of tRNA sequence, 5' part of tRNA and HHRz sequence, primer ai-H1 (Supplementary Figure S7, step 3). tRNA-HHRz chimera is located in the upstream of ibsC. To reduce misfolding of tRNA induced by irrelevant sequence, restriction enzyme site *Xba*I adjacent to T7 promoter was chose to insert tRNA-HHRz chimera sequence. And 3' end of tRNA is adjacent to T7 terminator.

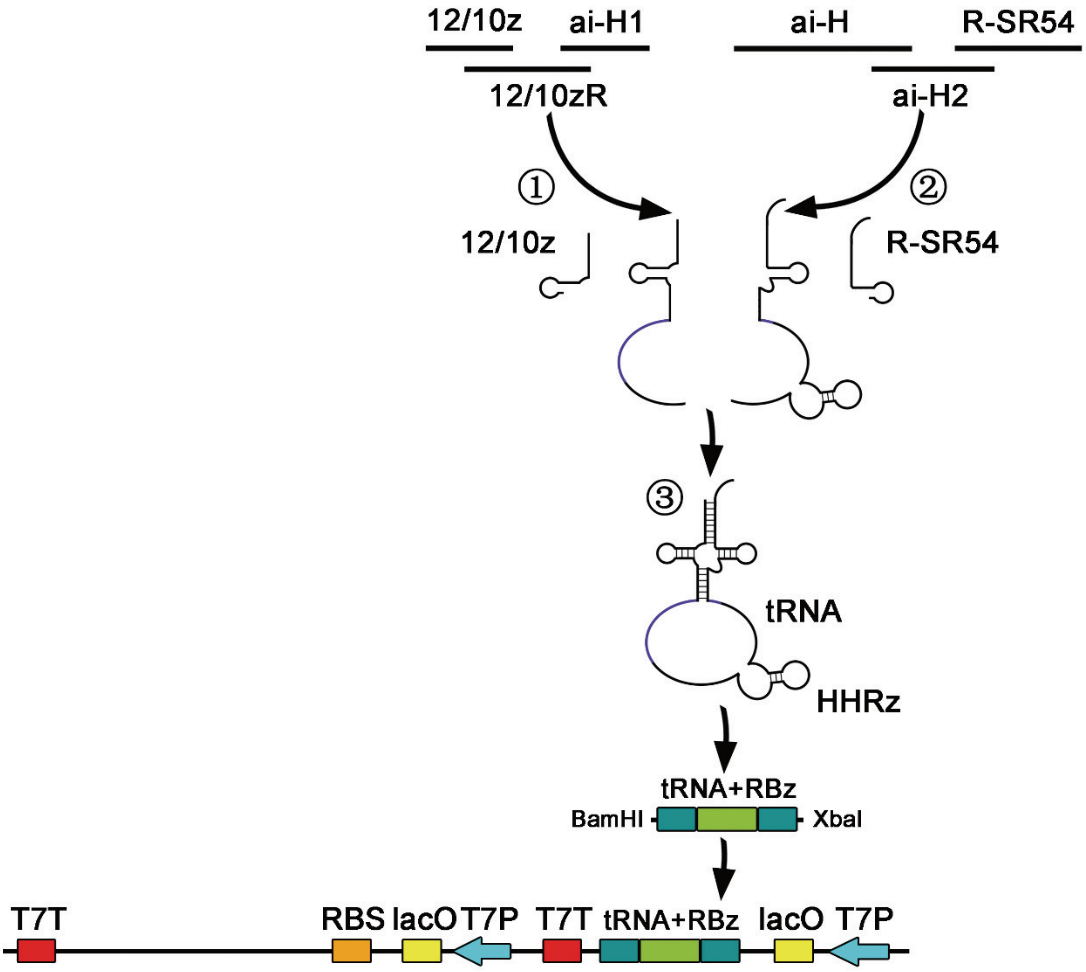

Supplementary Figure S7. Construction of plasmid for *trans*-acting HHRz selection. Step 1, 5'-part of tRNA with HHRz were amplified by primer 12/10z, 12.10zR and ai-H1; Step 2, 3'-part of tRNA with HHRz were amplified by primer ai-H, ai-H2 and R-SR54; Step 3, 5'- and 3'-part of tRNA with HHRz were amplified by an overlap PCR to generate full length tRNA and HHRz. This chimeric tRNA fragment was inserted into vector at *Bam*HI and *Xba*I site.

HHRz is constructed into tRNA by a linker. The nucleotide composition and length of linker connecting the HHRz to the tRNA may form structural constraint to decreased catalytic activity of tRNA-HHRz chimera compared with linear HHRz. Previous study has showed that 3' linkers would decrease the performace of HHRz, but 5' linkers could render the activity of HHRz[3]. Here, we choose CCACCAACAAAATCCA as 5' linker. CCACCAACAAAATCCA was modified into CCACCAACGAATTCCA to construct an *Eco*RI site. *Eco*RI restriction enzyme site was located in the middle of 5' linker of HHRz, meanwhile *Bam*HI site was devised in the 3' end of tRNA to render for the insertion of DNA pool effectively (Supplementary Figure S8).

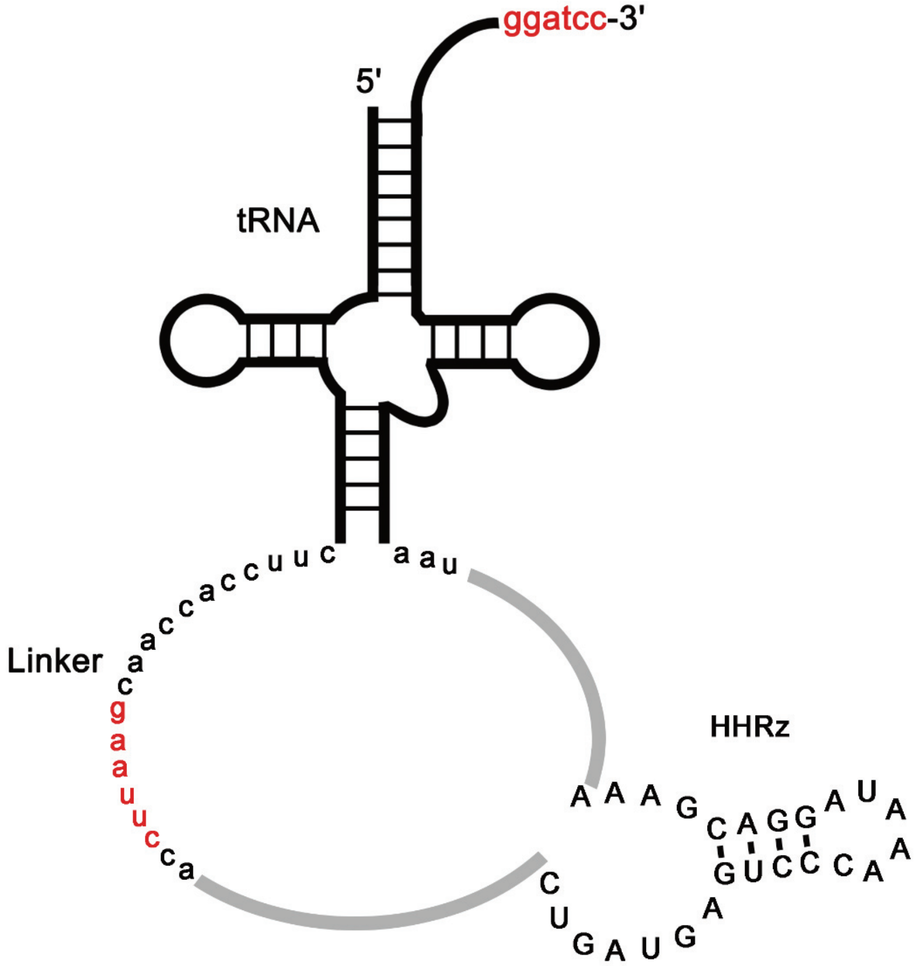

Supplementary Figure S8. Structure of tRNA and HHRz chimera. 5' end of HHRz was linked to tRNA by a sequence CCACCAACGAATTCCA. *Eco*RI restriction enzyme site is located in the middle of 5' linker of HHRz, and *Bam*HI site is devised in the 3' end of tRNA. Binding sequence of HHRz which are reverse complimentary to target sequence of substrate is labeled by grey. Sequence of tRNA is highlighted by dark. Restriction enzyme site are labeled by red.

S1.3.3 Relative higher expression of HHRz

If the expression of tRNA-HHRz chimera is much stronger than that of *ibsC*, HHRz could cleave mRNA of *ibsC* more drastically. Therefore, the lacO operator in the upstream of tRNA was deleted by a DNA fragment generated by primers EI5, Tac2 and T7X (Supplementary Figure S9, step 6). One lacO operator was devised into the upstream of reporter gene, as a brake to control the gene expression of *ibsC*. The expression of HHRz is not repressed by LacI, while expression of *ibsC* is regulated by LacI.

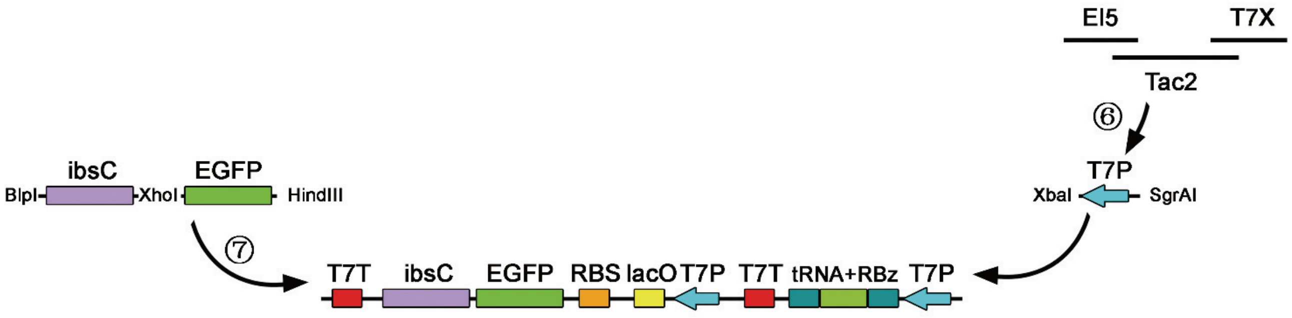

Supplementary Figure S9. Construction of plasmid for *trans*-acting HHRz selection. Step 6, EI5, Tac2 and T7X were used to amplified and inserted into vector at *Xba*I and *SgrA*I site to remove the lacO in the upstream of HHRz. Step 7, DNA sequence of *eGFP* and *ibsC* was inserted into recombinant plasmid at *Hind*III and *Blp*I.

Because IbsC is a small toxin protein (19 AARs), to slow the transcripton of *ibsC*, *egfp* gene was inserted in the upstream of *ibsC* at *Xho*I and *Hind*III site (Supplementary Figure S9, step 7). The eGFP was prepared synthetically by PCR using the primers GFP-5H and GFP-3mX (Supplementary Table S1) and DNA template peGFP. eGFP is fusion expressed in the N terminal of IbsC. The termination codon of *egfp* was removed to render for the normal translation of *ibsC*. PCR product of eGFP was subcloned into pTR7a1 vector in the *Hind*III and *Xho*I sites, giving rise to the pTR7a1Gm construct. Therefore, the relative expression amount of HHRz might be higher than reporter gene. To abolish the toxicity of IbsC, a linker sequence ATTCGGTGCGTCCTGGATTCA between *egfp* and *ibsC* was used to work as a target sequence of HHRz. The linker and *ibsC* sequence was prepared by primer ibsCb-5X and ibsC-3B, and inserted into vector at *Xho*I and *Blp*I site. Finally, a plasmid for intracellular selection of *trans*-acting HHRz was constructed and termed as pTR7a1Gmai.

S1.3.4 Intracellular selection of *trans*-cleaving HHRz

In this plasmid transformed cells, active *trans*-cleaving HHRz variants targeting the linker RNA could cleave the mRNA to interrupt its overexpression of IbsC, which render for the survival of *E. coli* on selection culture plate (Supplementary Figure S10), while inactive HHRz mutants could not (Supplementary Figure S10).

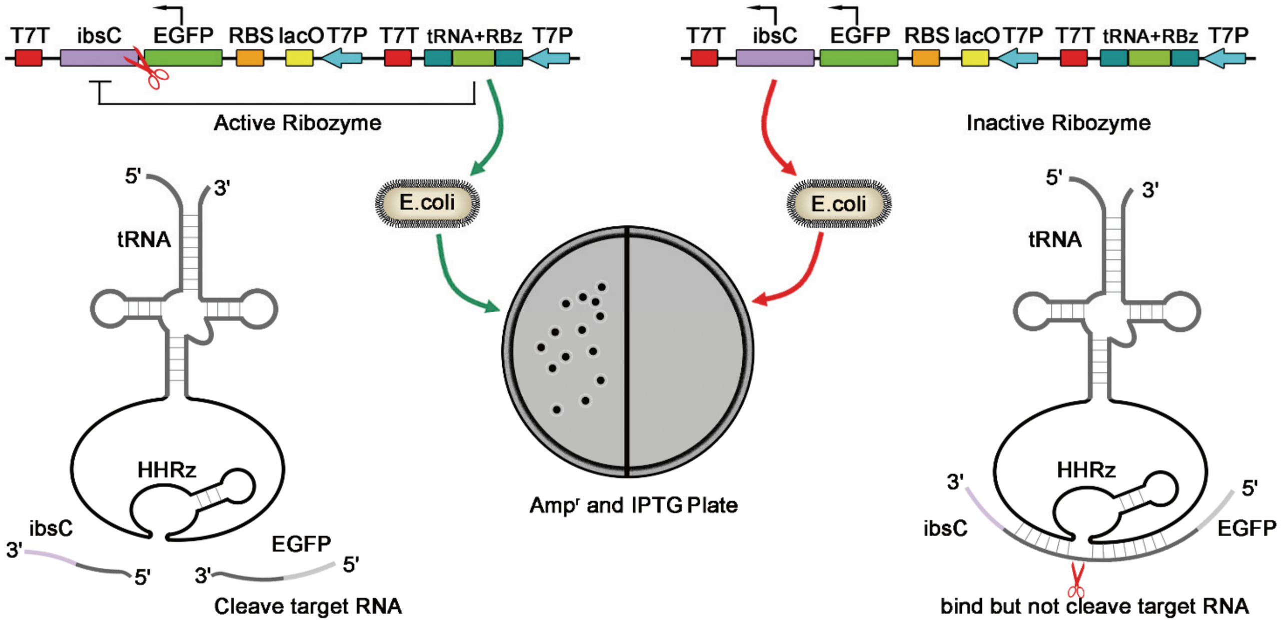

Supplementary Figure S10. Intracellular selection strategy of *trans*-cleaving HHRz via toxin protein. The transcription of *trans*-acting HHRz and *ibsC* is independent with each other. Active *trans*-cleaving HHRz cleave the mRNA of *ibsC* to render for the survival of *E. coli*, while *E. coli* including inactive HHRz mutants could not survive on the plate.

The selection dsDNA pools were prepared from PCR products of ai-HR1/ai-HR2, ai-H2 and R-SR54. We incorporated the DNA pools at *Eco*RI and *Bam*HI site into selection vector pTR7a1Gmai. A plasmid with inactive HHRz (pTR7a1Gmai-Hm) worked as negative control, and a vector with active HHRz (pTR7a1Gmai-H) was positive control. Recombinant selection vectors were electrotransformed into BL21(DE3) cells. 1h after recovery in 37 ºC and 150 rpm, all cells were collected and coated on resistant plate plus IPTG and incubated at 37 ºC overnight until *E. coli* with potential active HHRz mutants formed single colony.

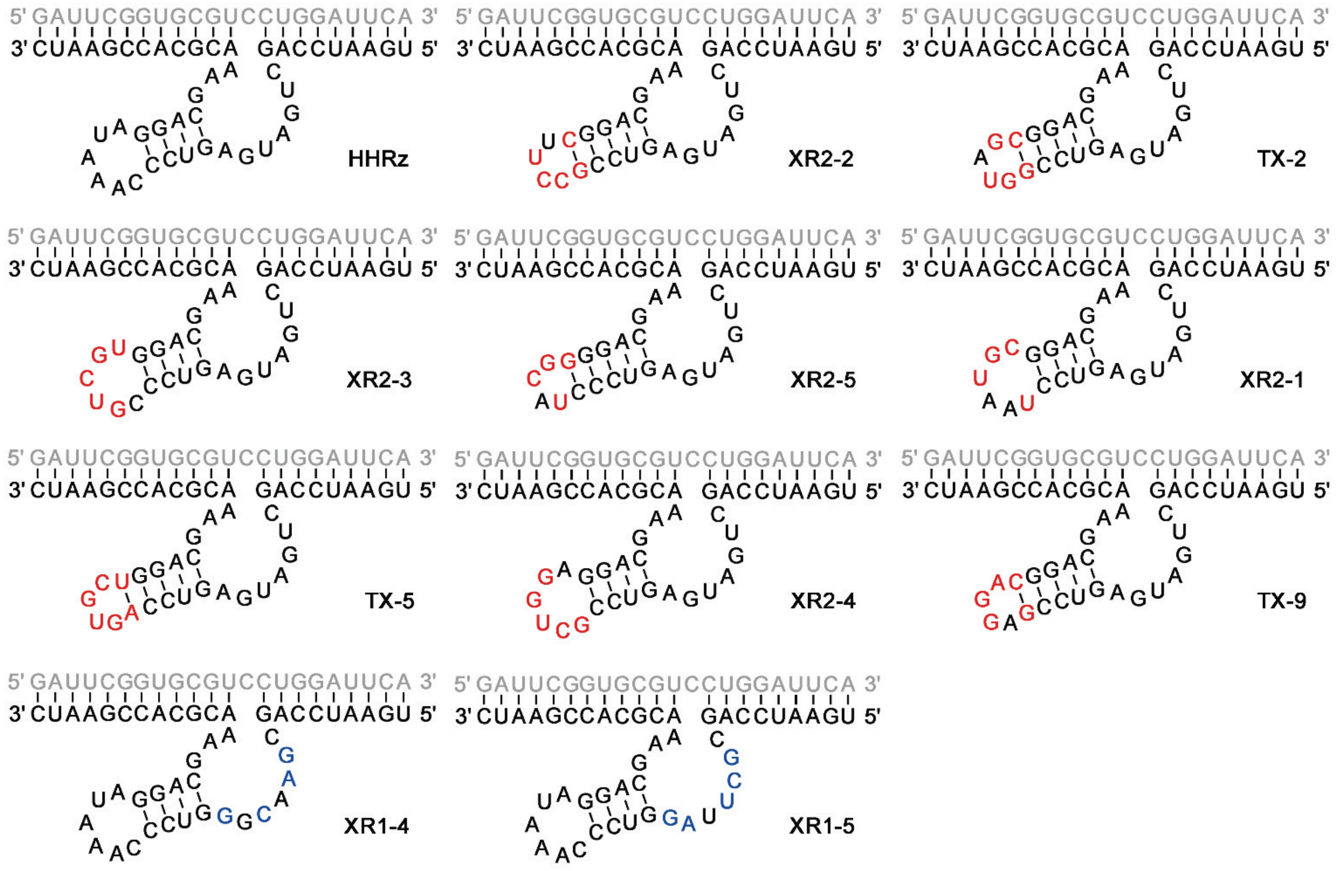

Supplementary Figure S11. Predicted structure of selected HHRz viriants by m-fold software. Modified nucleotides in Loop II in selected HHRzs were labeled as blue color. Mutant nucleotides in catalytic core in evolved HHRzs were labeled as red color.

After determination of clones by DNA sequencing, 10 *trans*-acting HHRz variants were generated by this intracellular selection method (Figure 2). As shown in Supplementary Figure S11, 8 of 10 mutants contained 4-6 nucleotide mutations in loop II. And 2 variants showed modifications in long catalytic core including 4-5 nucleotide mutations. All the modifications occurred in the random sequence region of selection pools. As showed in Supplementary Figure S11, regardless of nucleotides modifications, predicted structure of trans-acting HHRz mutants were similar with highly conserved structure of WT-HHRz. 5 variants exhibited extended stem II of 5 base pairs in length and relevant shorter loop II of 4 nucleotides in length.

S1.4 *In vitro* cleavage expriments of WT-HHRz and mutants

The cleaved activity of HHRz mutants was evaluated by *in vitro* isotope-labeled experiments under 0.5 mM Mg(II) condition. HHRz variants TX-2, TX-5 and TX-9 gave much better results than WT-HHRz in physiological conditions.

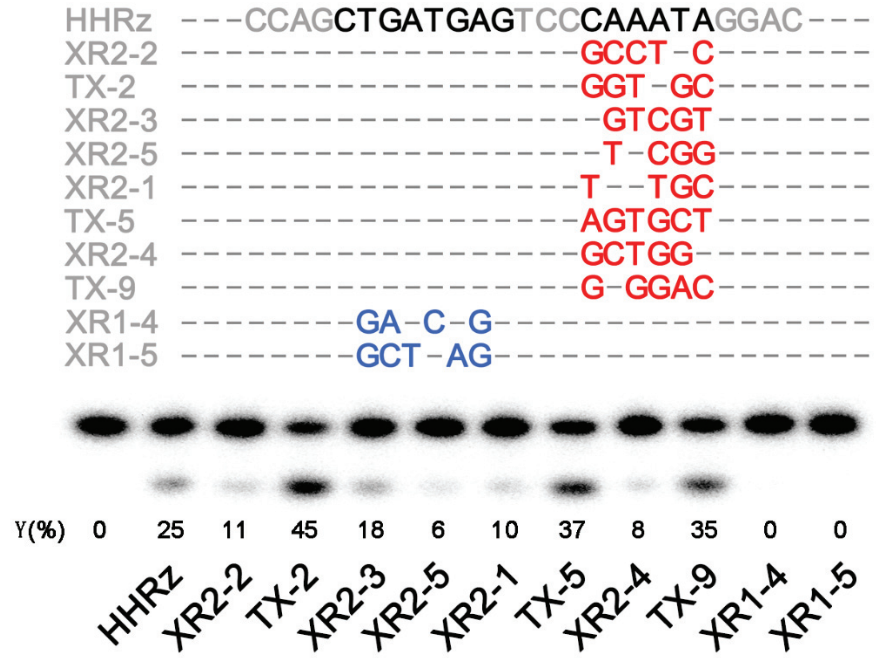

Supplementary Figure S12. *Trans*-cleavage reactions were performed for 4h in 0.5mM $MgCl_2$ at 37 ºC in 50mM Tris-HCl (pH 7.4), 200 mM NaCl and 10 mM $MgCl_2$. Lane 1, 5'-p^{32} labeled RNA substrate. Lane 2, cleavage of HHRz. Lane 3~12, cleavage of HHRz various variations.

S1.5 Definition of intracellular cleavage efficiency of selected HHRz mutants by double fluorescent protein system

S1.4.1 Vector construction of *trans*-cleaving HHRz targeted mCherry

Intracellular selection based on IbsC toxin has been proved to be a useful tool to generate trans-cleaving HHRz variants. This method could distinguish active and inactive effectively. But it is hard to determine the capacity of HHRz quantitively intracellular. Therefore, we design another strategy to define the cleavage capacity of HHRz. Red fluorescent protein gene, *mCherry*, was prepare by primer RI-5X and R-3B to replace *ibsC* as a reporter gene here. Active HHRz could cleave the mRNA of *mCherry* to reduce the fluorescence of *E. coli* (Supplementary Figure S12B), while inactive HHRz didn't alter bacterial fluorescence intensity (Supplementary Figure S12A). The translation of *mCherry* in different cells is not always same as each other. Here we used egfp as an internal reference to normalize the expression of *mCherry*. EGFP is in the upstream of mCherry and its expression doesn't be influenced by HHRz (Supplementary Figure S12B).

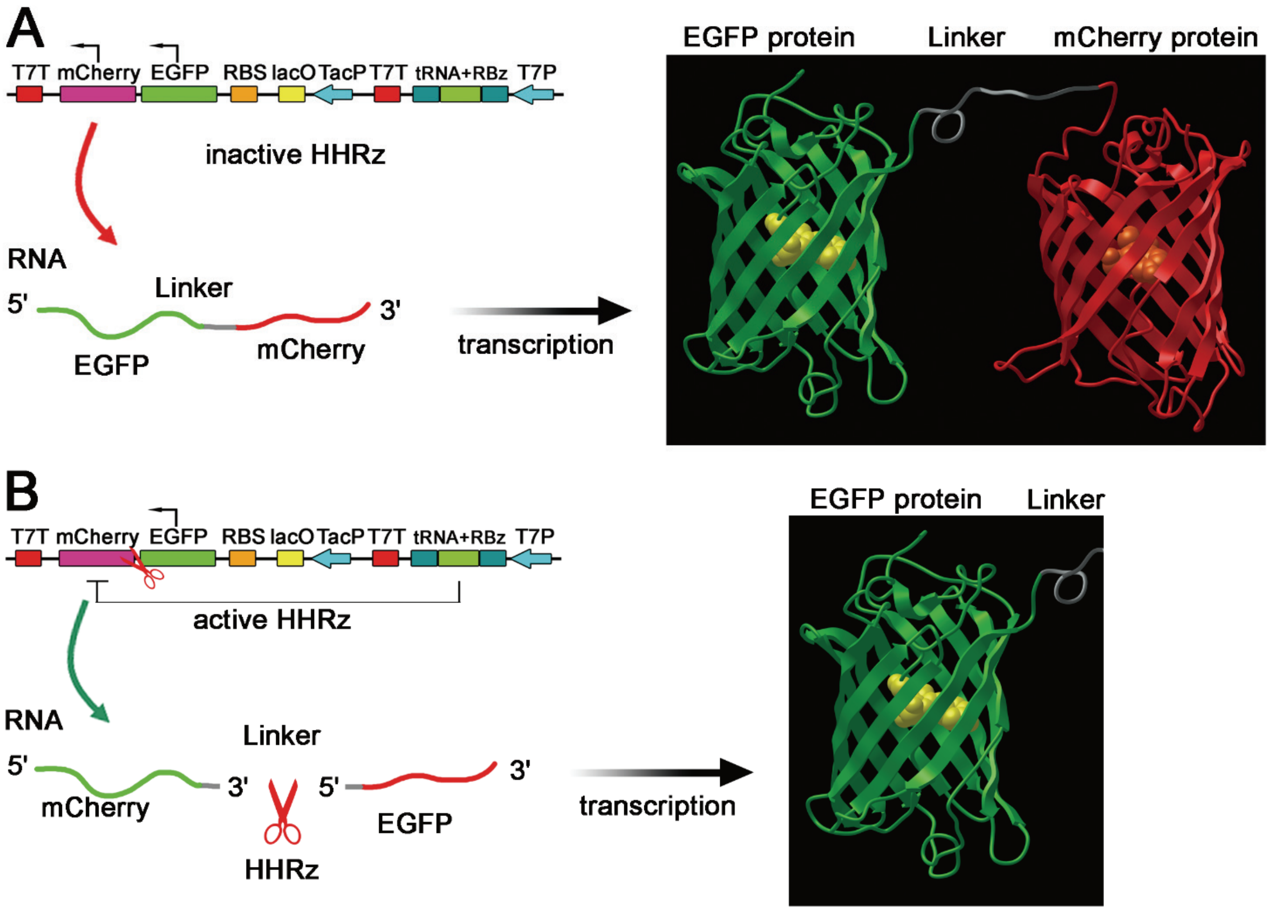

Supplementary Figure S13. Intracellular defining of *trans*-cleaving HHRz mutants *via* dual fluorescent proteins. A, inactive HHRz could not disturb the normal expression of eGFP and mCHerry. B, active *trans*-acting HHRz cleave the linker between *mCherry* and *egfp* to abolish the expression of mCherry but not interfere that of eGFP.

S1.4.2 Expression of HHRz and fluorescent protein by different promoters

To cleave substrate RNA thoroughly, the relative amount of HHRz should be much higher than substrate. T7 and Tac promoter are both strong promoter, but the initiation efficiency of T7 promoter is much higher than that of Tac promoter. And the RNA polymerase used by T7 and Tac are different. Therefore, the transcription of HHRz was initiated by T7 promoter.

But, the expression of fluorescent protein was initiated by Tac promoter. The competitive inhibition of transcription initiated by T7 and Tac promoter could be reduced to largest extent. To increase the expression efficiency, tandem dual Tac promoter (TTGACAATTAATCATCCGGCTCGTATAATGCGAACAGAAAGTTTGACAATTAATCATCCGGCTCGTATAATG) was constructed in the upstream of fluorescent protein gene. The primers T7TFS, T7TFL.1, iTac-1R, iTac-2, iTac-3R and iT7-3aR (Supplementary Table S1) were used to prepare dual Tac promoter dsDNA. PCR product was cloned into pTR7a1GmaR vector in *Bam*HI and *Hin*dIII site to yield pTRDTa1GmaR construct.

S1.4.3 Intracellular definition of *trans*-cleaving HHRz targeted linker sequence

We have obtained 10 HHRz mutants using toxin as reporter gene. To quantificationally evaluate the intracellular efficiency of these HHRz mutants, PCR products containing HHRz variants, WT-HHRz and HHRzm were constructed into pTRDTa1GmaR. Recombinant vectors were transformed into BL21(DE3) and coated on resistant plate for overnight. More than three independent monoclones of each HHRz mutants were cultivated in 1ml LB at 37 ºC and 250 rpm to reach logarithmic phase (OD600=0.3), then induced by IPTG at 37 ºC and 150 rpm for 18h. Cells were precipitated in 4000 rpm for 5 mins and supernatant were removed. Cell precipitates were washed with PBS twice to remove LB medium and dead cells, and then resuspended with PBS. The PBS resuspended cells were analyzed by FCM (excitation 488nm, emission 520nm (FL1) for EGFP; excitation 561nm, emission 610nm (FL11) for mCherry; Moflo XDP, Beckman Coulter). As shown in (Supplementary Figure S13), all the HHRz mutants showed decreased red fluorescence compared with HHRzm. The fluorescence of EGFP was relatively constant. Especially, TX2, TX5 and TX9 showed increased relative cleavage efficiency (2.26, 1.40 and 1.63-fold higher than that of WT-HHRz, respectively) (Supplementary Figure S14). These results suggested that this dual fluorescent proteins system could determine the intracellular cleavage efficiency of selected HHRzs.

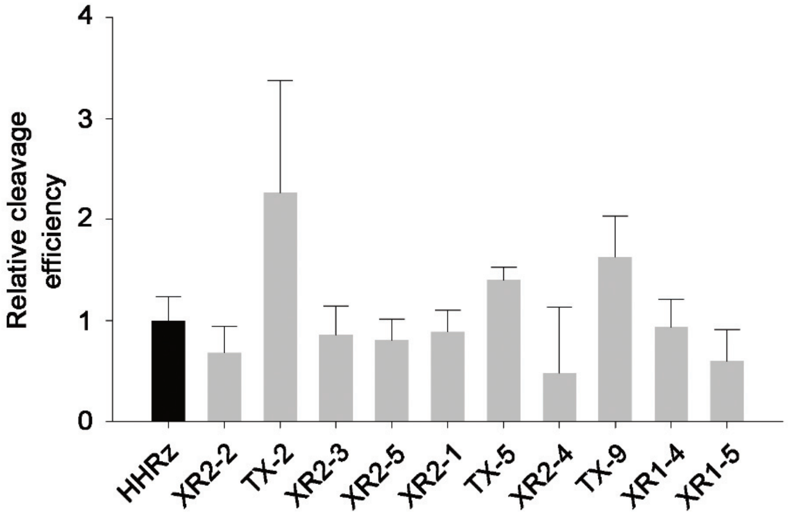

Supplementary Figure S14. Relative cleavage efficiency of HHRz variants selected by IbsC toxin system. BL21(DE3) competent cells transformed by vectors with *trans*-acting HHRzm, HHRz, and selected mutants were coated on the ampicillin plate overnight. At least three monoclones were picked up into 1 ml LB and cultured to 0.3 OD600 following induction by IPTG for 18h. Cells were collected and the fluorescence of cells were analyzed by FCM. Relative cleavage efficiency was defined as follow: Relative cleavage efficiency=(RHm-RX)/(RHm-RH); RHm=mCherry/EGFP ratio of HHRzm, RH=mCherry/EGFP ratio of HHRz, RX=mCherry/EGFP ratio of HHRz variants; Relative cleavage efficiency of HHRz was identified as 1.

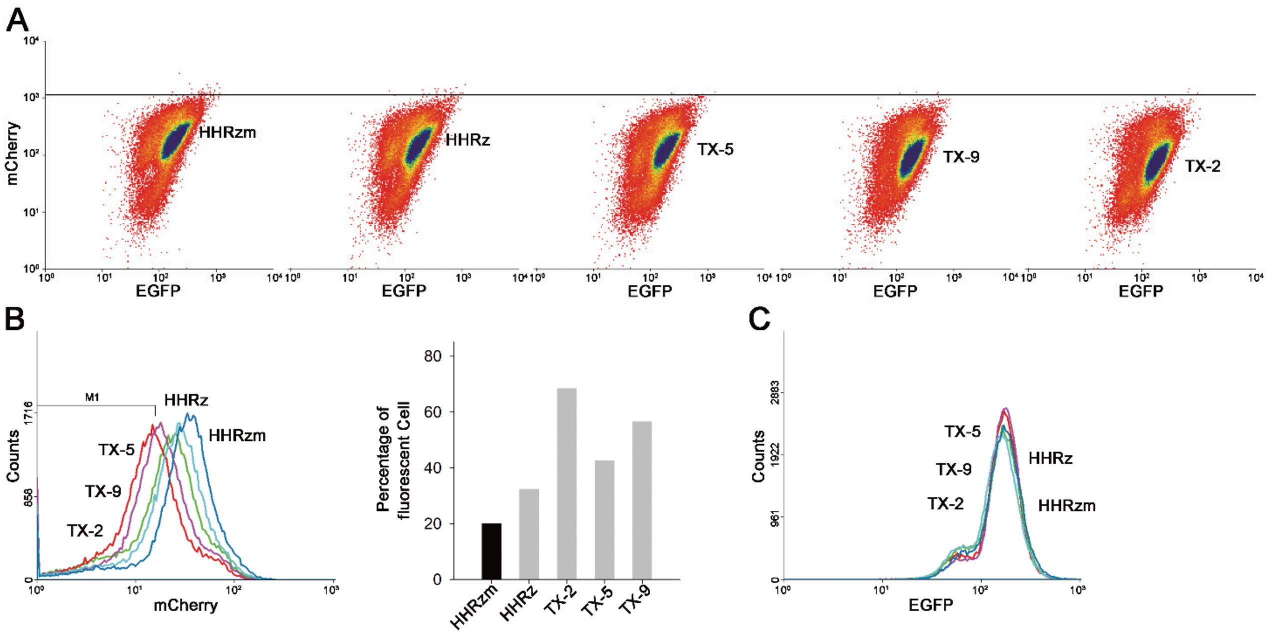

Supplementary Figure S15. Intracellular definition of *trans*-cleaving HHRz targeted linker sequence: A, FCM dot plot presenting the fluorescence distribution of BL21(DE3) transformed with different HHRzm, HHRz and three selected HHRz mutants which showed decreased florescence compared with WT HHRz. B, FCM histogram showing one of the representative result of red fluorescent shift of HHRz variants. Binding sequences of HHRz were designed according to complementary target sequence of linker, GAUUCGGUGCGUCCUGGAUUCA, and cleavage site was labeled as red color. C, FCM histogram showing one of the representative result of green fluorescent shift of HHRz variants.

S1.4.4 Selection of target sites on mCherry mRNA

To further test the generality of these selected HHRz variants, we choose another site of mCherry to be targeted by HHRz mutants. Open reading frame (ORF) of *mCherry* is 708 base pairs in length. There are many potential cleavage sites in the linear sequence of RNA. However due to the steric hindrance formed by single strand RNA folding, there may exist only a few sites within mRNA containing tertiary structure those are accessible to hybridization. It is necessary to identify the optimized cleavage site of *mCherry* for HHRz. We have compared four potential sites (260, 374, 467 and 533) on *mCherry*.

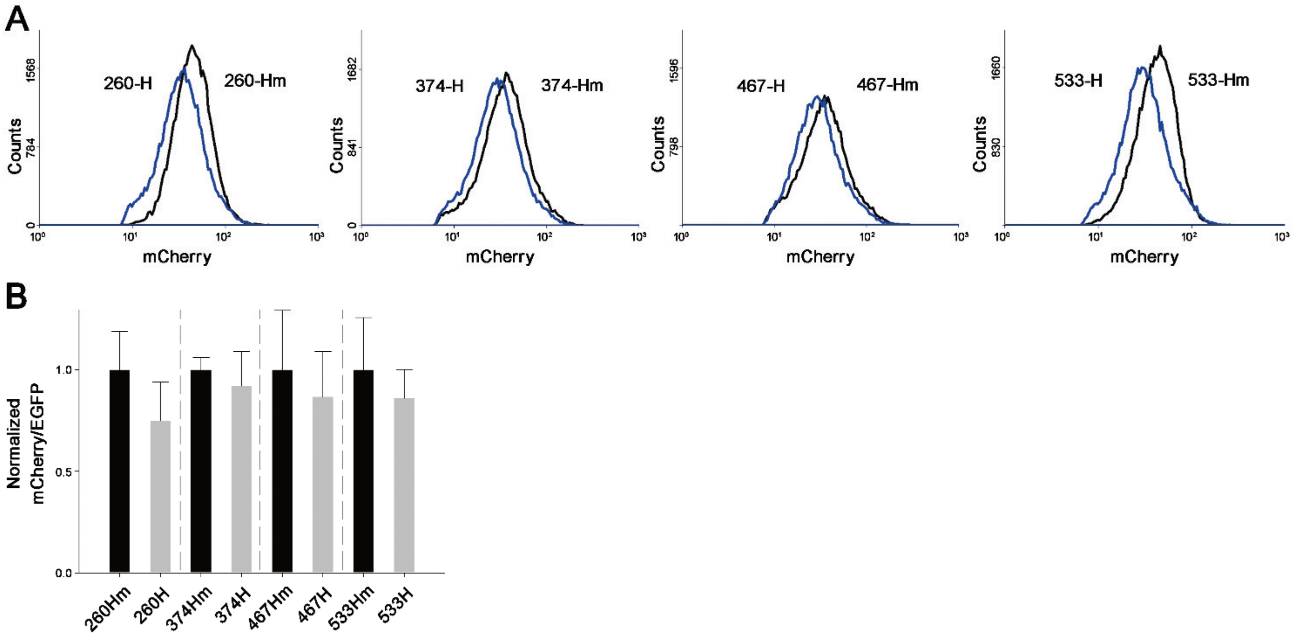

Supplementary Figure S16. Selection of optimized cleaved site of HHRz on mCherry. A, fluorescence drift of HHRz which cleave mCherry in different sites compared with HHRzm. B, normalized fluorescence of HHRz in different cleavage sites. Cells were transformed by plasmids containing HHRzs that cleave mRNA of mCherry at site 260, 374, 467 and 533 respectively. Vector including inactive HHRzm at each site was used as negative control. Transformed cells were coated on the resistant plate overnight and monoclones of each vector were inoculated in 1ml LB medium and induced by IPTG (final concentration 1mM) when OD600 reached 0.3. Cells were centrifuged and analyzed by FCM. The results show the normalized fluorescence ration between EGFP and mCherry. The mCherry/EGFP ratio of HHRzm (Hm) of each cleavage site was identified as 100%.

The binding sequences of HHRz were modified according to the given site of *mCherry*. DNA products were amplified by primer R-SR54, R260Sa or R260Sam and R260Sb (Supplementary Table S1) into pTRDTa1GmR vector at *Eco*RI and *Bam*HI site, constructing pTRDTa1GmR-260H and pTRDTa1GmR-260Hm. Primers R-SR54, R374Sa/R374Sam and R374Sb (Supplementary Table S1) were used to constructed pTRDTa1GmR-374H and pTRDTa1GmR-374Hm; pTRDTa1GmR-467H and pTRDTa1GmR-467Hm were prepared by primer R-SR54, R467Sa/R467Sam and R467Sb (Supplementary Table S1); Primers R-SR54, R533Sa/R533Sam and R533Sb (Supplementary Table S1) were applied to generate pTRDTa1GmR-533H and pTRDTa1GmR-533Hm. Plasmid pTRDTa1GmR-X-Hm (X=260, 374, 467 or 533) represent the long catalytic core of HHRz was modified to loss of cleavage capacity and work as a control.

E. coli transformed by recombinant vectors were cultivated to logarithmic phase (OD600=0.3) and then induced for 18h by IPTG to express HHRz and fluorescent proteins. The fluorescence of *E. coli* was confirmed by FCM. As shown in Supplementary Figure S15A, *E. coli* with HHRz presented decreased fluorescent intensity in contrast to that with HHRzm, which revealed that active HHRz could down-regulate the expression of mCherry by RNA cleavage. The cells with active HHRz showed decreased fluorescent intensity (24.96%, 7.90%, 13.22%, and 13.90% at site 260, 347, 467 and 533 respectively) in contrast to that of HHRzm. Site 260 and 533 showed declined fluorescence to the greatest extent (Supplementary Figure S15 B). So, we concentrate on the optimized cleaved site of mCherry at 260 and 533.

S1.4.5 Intracellular definition of *trans*-cleaving HHRz targeted 260 and 533 of mCherry

As shown results of targeted linker sequence between EGFP and mCherry, selected TX2, TX5 and TX9 also showed good intracellular performance at 260 (Supplementary Figure S16) and 533 (Supplementary Figure S17) than WT-HHRz. These observations demonstrated that the cleavage efficiency of HHRz mutants could be well determined by the dual fluorescence proteins system.

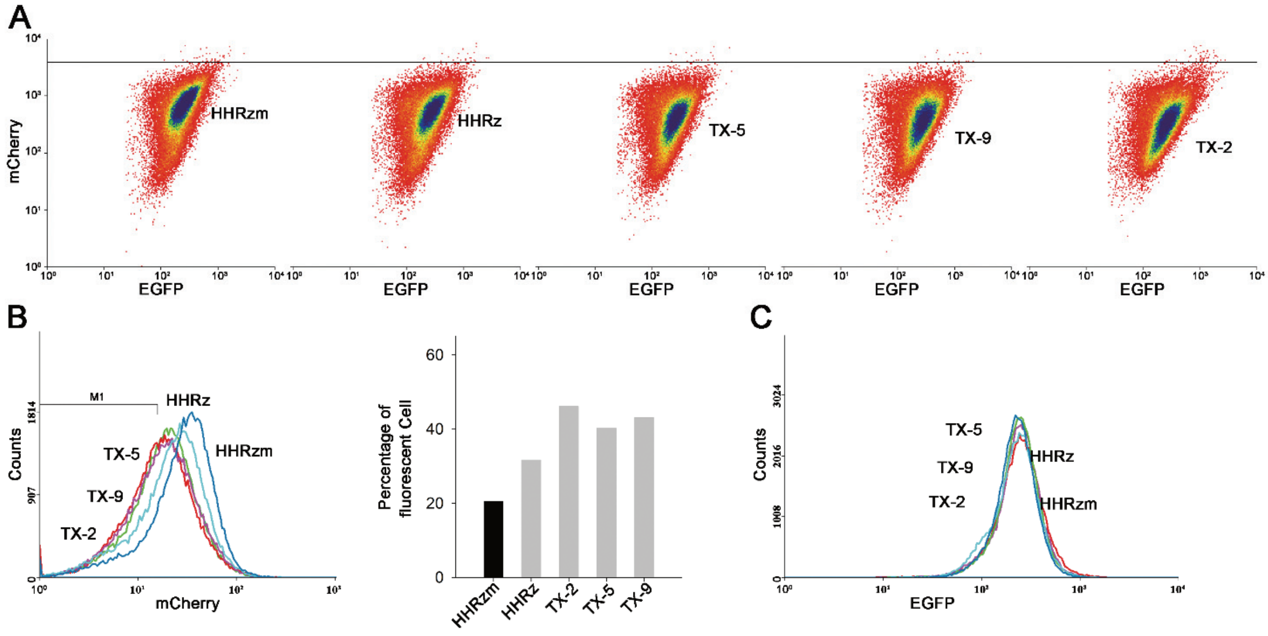

Supplementary Figure S17. Intracellular definition of *trans*-cleaving HHRz targeted 260 of *mCherry*: A, FCM dot plot presenting the fluorescence distribution of BL21(DE3) transformed with different HHRzm, HHRz and three selected HHRz mutants which showed decreased florescence compared with WT HHRz. B, FCM histogram showing one of the representative result of red fluorescent shift of HHRz variants. Binding sequences of HHRz were designed according to complementary target sequence of nucleotide 260 of mCherry, CCCCGACUACUUGAAGCUGUC. C, FCM histogram showing one of the representative result of green fluorescent shift of HHRz variants.

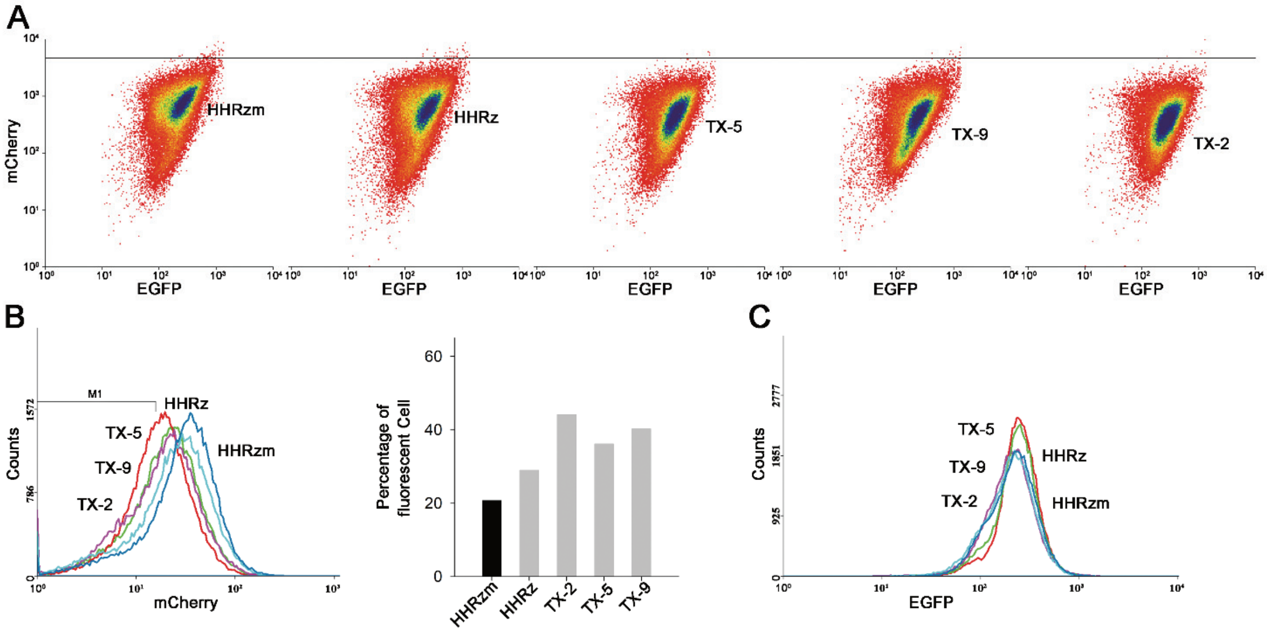

Supplementary Figure S18. Intracellular definition of *trans*-cleaving HHRz targeted 533 of *mCherry*: A, FCM dot plot presenting the fluorescence distribution of BL21(DE3) transformed with different HHRzm, HHRz and three selected HHRz mutants which showed decreased florescence compared with WT HHRz. B, FCM histogram showing one of the representative result of red fluorescent shift of HHRz variants. Binding sequences of HHRz were designed according to complementary target sequence of nucleotide 533 of mCherry, CGGCCACUACGACGCUGAGGU. C, FCM histogram showing one of the representative result of green fluorescent shift of HHRz variants.

S1.6 Definition of intracellular cleavage efficiency of selected HHRz mutants at RNA level

S1.5.1 Vector construction of *trans*-cleaving HHRz targeted Spinach

Our above selection methods generated active HHRz mutants according to the activity variations of reporter gene in protein level. However, the substrate of HHRz is RNA but not protein. Therefore, here we use a RNA termed Spinach as a reporter to reveal the intracellular activity of HHRz in RNA level. Spinach is a RNA molecular which could bind with a small chemical molecular DFHBI to form RNA-DFHBI complex. This complex could be induced green fluorescence in the excitation of laser [4]. Active HHRz would cleave the RNA of Spinach and reduce the bacterial fluorescence (Figure 3).

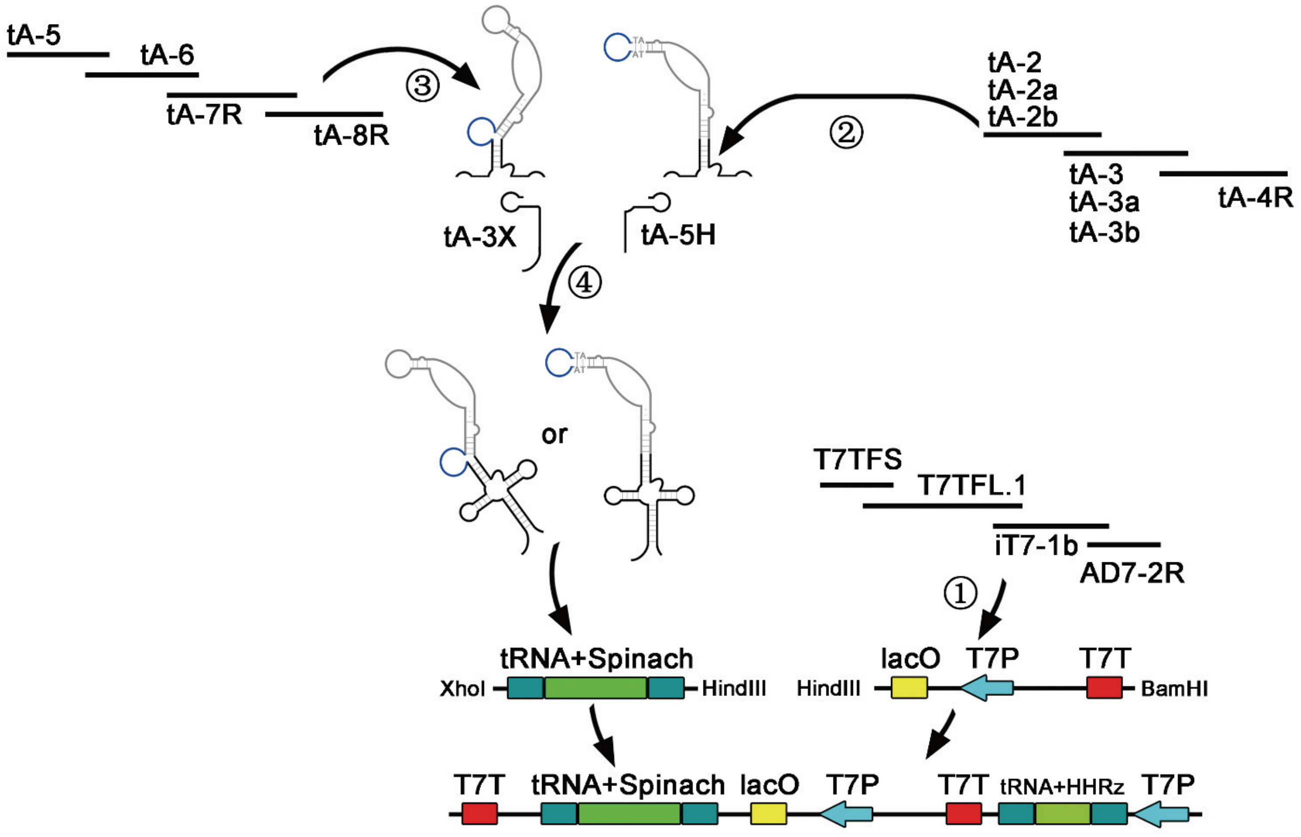

Supplementary Figure S19.Vector construction for intracellular defining of HHRz's activity by Spinach. Step 1, to remove the RBS before Spinach, primer T7TFS, T7TFL.1, iT7-1b and AD7-2R were amplified by an overlap PCR and inserted into vector at *Hin*dIII and *Bam*HI site. Step 2 and 3, primers were used to prepare the main sequence of chimeric tRNA with Spinach. Full length chimeric tRNA were amplified by tA-5H and tA-3X using PCR products of step 2 or 3 as templates and constructed into plasmid at *Xho*I and *Hin*dIII site.

The Spinach was amplified by PCR using the five or six primers to generate full length dsDNA flanked by *Hin*dIII and *Xho*I sites (Supplementary Figure S18). Our initial selection based on the cleavage of RNA of *ibsC*, sequence of Spinach was replaced with DNA sequence of *ibsC* (GTACTGTTACTCATAAGTTTCAGCGCT) to test the cleaving activity of HHRz on RNA of *ibsC*. Previous studies have certified that substitution of loop II of Spinach don't affect its property for fluorescence inducing property [5,6]. There are four base pairs in the stem region of replaceable loop for Spinach. Replacement of this loop may affect the folding of Spinach. Therefore, we artificially prolong the stem length to 4 base pairs, 5 base pairs and 6 base pairs (Supplementary Figure S19A). The DNA fragments contain 4 base pairs stem was prepared by primers tA2, tA3 and tA-4R; DNA with 5 base pairs stem was produced by primers tA2a, tA3a and tA-4R; primers tA2b, tA3b and tA-4R were used to amplify the DNA sequence including 6 base pairs stem (Supplementary Figure S18, step 2). At the same time, insertion of DNA sequence of ibsC as 5'-linker between tRNA and Spinach was yielded by primers tA-5, tA-6, tA-7R and tA-8R (Supplementary Figure S18, step 3). Full length chimeras of tRNA and Spinach were further amplified by primers tA-5H and tA-3X (Supplementary Figure S18, step 4) and subcloned into vector pTR7a1i at *Xho*I and *Hin*dIII to construct recombinant vectors pTR7a1A1, pTR7a1A2, pTR7a1A3 and pTR7a1A4. Spinach is RNA molecular with capacity for binding

with DFHBI to induce fluorescence. So RBS region before Spinach was deleted using primers T7TFS, T7TFL.1, iT7-1b and AD7-2R at restriction enzyme *Hin*dIII and *Bam*HI site (Supplementary Figure S18, step 1).

S1.5.2 Intracellular definition of *trans*-cleaving HHRz targeted various Spinach and tRNA chimeras

DNA fragments containing HHRz were prepared by three primers: i42-H1, i42-H/i42-Hm/i42-TX-2/i42-TX-5/i42-TX-9 and i42-H2R. Active HHRz and tRNA chimera was cloned into these vectors to work as positive control to identify whether the HHRz could cleave the RNA of Spinach. Inactive HHRzm was a negative control to assess the effect of RNA binding on cleavage. Another plasmid containing unmodified tRNA and Spinach, termed as pAp, was a negative control to evaluate the effect of the loop replacement on property of Spinach. These recombinant vectors were transformed into BL21(DE3) competent cell. *E. coli* transformed by recombinant vectors were cultivated to logarithmic phase (OD600=0.3) and induced for 4h by IPTG to transcribe RNA of HHRz and Spinach.

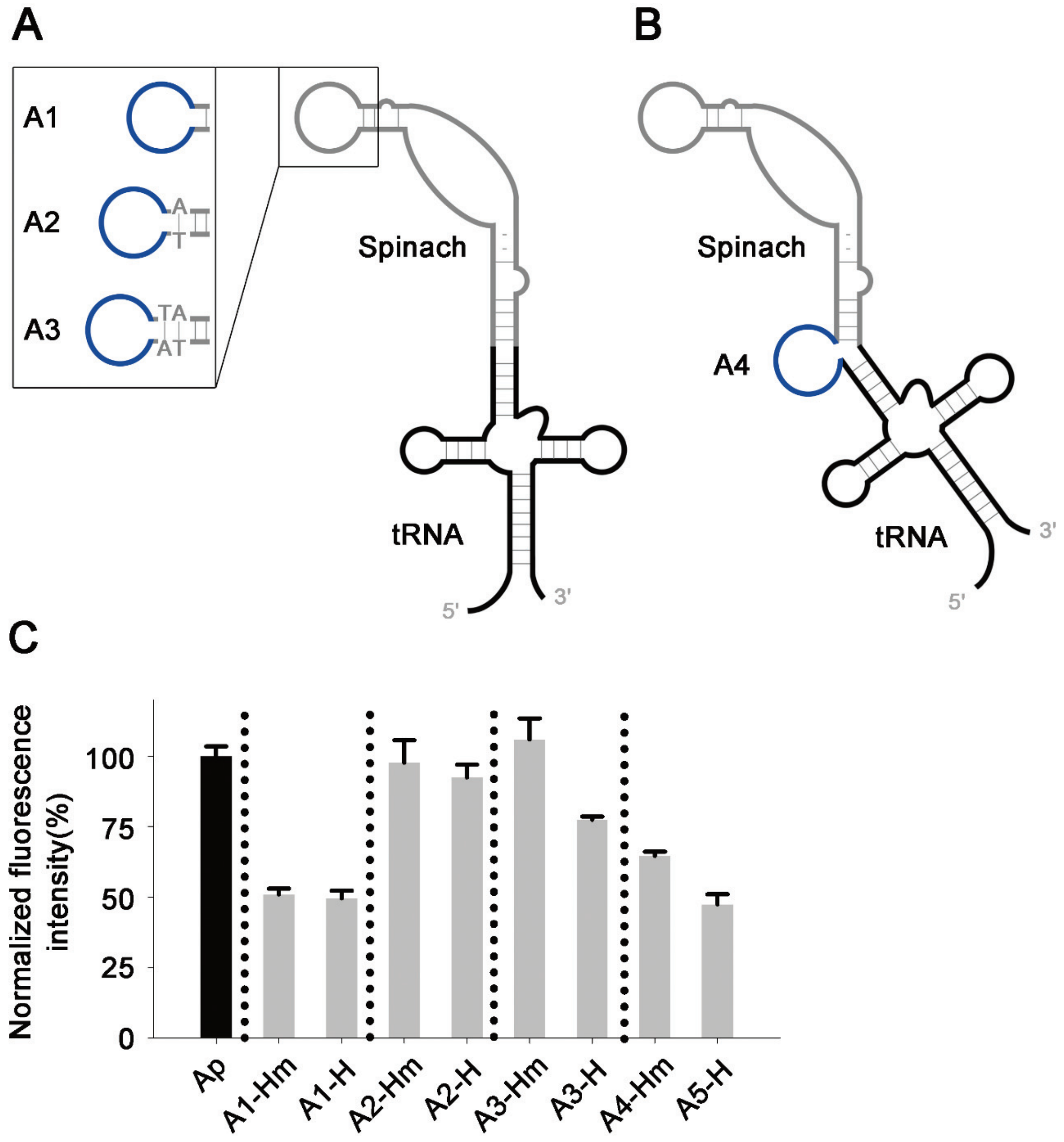

Supplementary Figure S20. Selection of optimized cleaved site of HHRz on Spinach. A, sequence in loop adjacent to steam P2.2 of Spinach were replaced by partial sequence of ibsC. TR7a1A1, TR7a1A1 and TR7a1A1 contained P2.2 in different length. B, partial sequence of ibsC was inserted as 5'-linker between tRNA and Spinach to construct vectors TR7a1A4. C, Cells were transformed by plasmids pTR7a1A1-H, pTR7a1A2-H, pTR7a1A3-H, pTR7a1A4-H which containing active HHRz. Vector including inactive HHRzm at each site was used as negative control and plasmid without HHRzm or HHRz (termed as Ap) was designed as positive control. Transformed cells were coated on the resistant plate overnight and monoclones of each vector were inoculated in 5ml LB medium and induced by IPTG (final concentration 1mM) at 37 ºC and 150 rpm for 6h when OD600 of *E. coli* reached 0.3. Cells were pelleted and

resuspended with 200µl PBS. Resuspended cells were incubated with DFHBI (final concentration 200µM) at 37 ºC for 1h. Then the fluorescence of incubated cells with various HHRz variants were evaluated by FCM. The fluorescence of Ap was identified as 100%.

As shown in Supplementary Figure S19, replacement of loop in constructs A2Hm and A3Hm don't modify the fluorescence intensity of Spinach compared with Ap which contain original sequence of Spinach. However, fluorescence intensity of A1Hm and A4Hm showed 49.00% and 35.43% decrease in contrast to Ap, which demonstrate these constructs would affect the folding of Spinach and lead to the decreased fluorescence. The fluorescence intensity of A1H and A2H is similar with A1Hm and A2Hm, respectively. A3H and A4H showed decreased fluorescence compared with A3Hm and A4Hm. The fluorescence of A3H and A4H was 26.95% and 26.91% lower than that of A3Hm and A4Hm, respectively. Taking account of the A4 construct would reduce the fluorescent intensity of Spinach, our following experiments focused on the vector pTR7a1A3.

As shown in Supplementary Figure S20, the fluorescent population of transformed cells was significantly decreased in presence of active ribozymes when compared to that of inactive HHRzm.

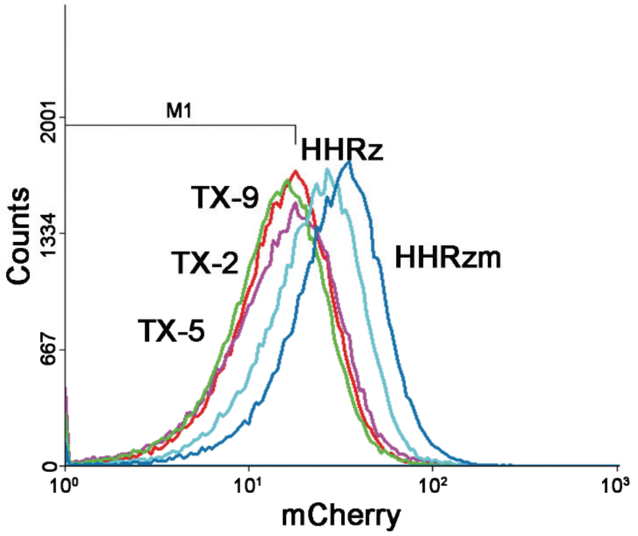

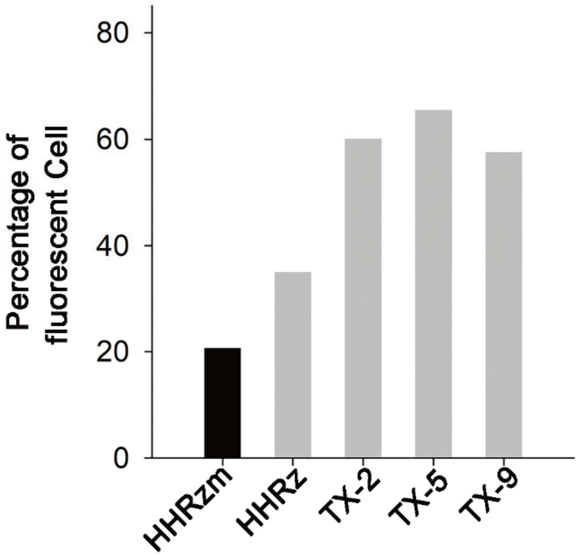

Supplementary Figure 21. Intracellular characterization of *trans*-cleaving by Spinach. FCM histogram showing the green fluorescent shift of HHRz variants.

S1.7 Intracellular cleavage efficiency of HHRz variants in human cancer cells

S1.6.1 Eukaryotic vector construction of *trans*-cleaving HHRz

Selected HHRz mutants were cloned in the eukaryotic vector pmCherry-C1. To avoid the expression of HHRz to disturb that of *mCherry*, HHRz sequences were reversely inserted into downstream of *mCHerry*. Various HHRz mutants were PCR amplified using five primers (Supplementary Figure S21, step 3 and 4): t1F, t2R, Rt260F1Hm/Rt260F1H/Rt260F1X2/Rt260F1X5/Rt260F1X9, Rt260F2 and RtH-4R for *mCherry* (Supplementary Table S1). The vectors targeted other genes were prepared as follow: *BCL2* (t1F, t2R, Lt1032F1Hm/Lt1032F1H/Lt1032F1X2/Lt1032F1X5/Lt1032F1X9, Lt1032F2 and RtH-4R); *GAPDH*, (t1F, t2R, Gt369F1Hm/Gt369F1H/Gt369F1X2/Gt369F1X5/Gt369F1X9, Gt369F2 and RtH-4R). t1F contained *Sal*I site and RtH-4R possessed six continuous A as U6 terminator and *Hin*dIII site. These resulting DNA fragments of HHRz expression were reversely inserted in the downstream of *mCherry* gene. To stop the transcription of *mCherry* correctly, a polyA sequence was subcloned into the plasmid at *Xho*I and *Hin*dIII site by product of two prime: bGH -5X and bGH -3H (Supplementary Figure S21, step 5).

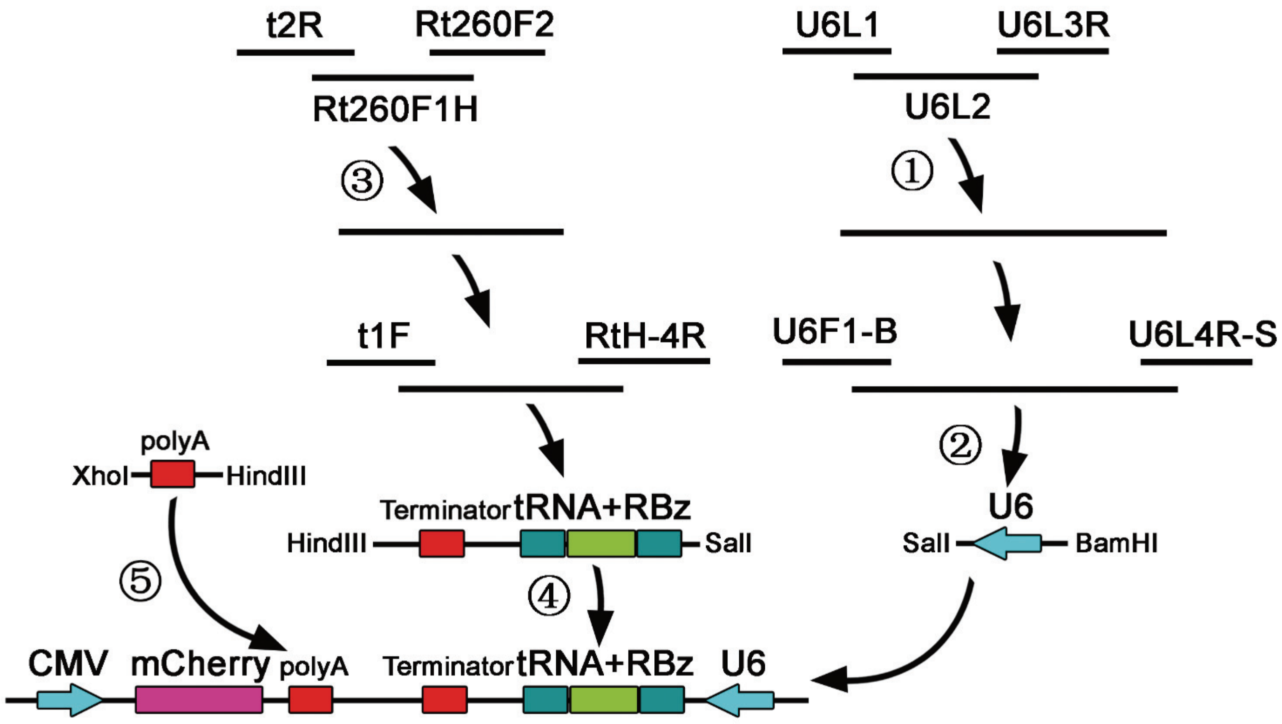

Supplementary Figure S22. Eukaryotic vector construction of *trans*-cleaving HHRz targeted *mCherry*. Full length U6 promoter was prepared by two round of PCR: Step 1, U6L1, U6L2 and U6L3R were used in the first round PCR to yield a DNA template of the second round PCR; Step 2, U6F1-B, U6L4R-S and PCR products of first round were used to yield full length DNA products that then cloned into plasmid at *Sal*I and *Bam*HI site. HHRz was amplified by five primers shown in step 3 and 4, then cloned into plasmid at *Hin*dIII and *Sal*I site. Step 5, a polyA sequence was subcloned into the plasmid at *Xho*I and *Hin*dIII site by product of two prime: bGH -5X and bGH -3H.

Full-length U6 promoter containing upstream promoter elements, TATA box and the transcription start site was prepared by five primers: U6F1-B, U6L1, U6L2, U6L3R and U6L4R-S (Supplementary Figure S21, step 1 and 2). The PCR product was inserted into plasmid at *Bam*HI and *Sal*I site. Colonies were isolated and correct clones were confirmed by restriction enzyme analysis. Clones expressing HHRz mutants were then sequenced.

S1.6.2 Intracellular cleavage efficiency of HHRz variants target mCherry in *Hela* cells

An active ribozyme (WT-HHRz) was also cloned in pmCherry-C1 to serve as a positive control. In addition, an inactive ribozyme (HHRzm) was cloned in pmCherry-C1 to serve as a negative control. Recombinant vectors containing HHRz, HHRzm, TX-2, TX-5 and TX-9 were transfected into *Hela* cancer cells by Lipofactmin2000. After 48h post-transfection, cells were collected and fluorescence was tested by FCM. As shown in Supplementary Figure S22, TX-2 in all HHRz variants provided the best suppression of targeted mCherry gene. TX5 and TX9 also suppress the expression of mCherry more effectively than WT-HHRz.

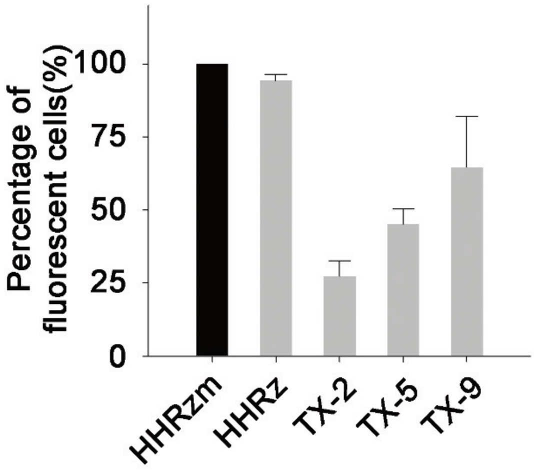

Supplementary Figure S23. Intracellular cleavage efficiency of HHRz variants target mCherry in *Hela* cells. *Hela* cells were transfected with recombinant constructs containing HHRz, HHRzm and variants. 48 h post-transfection, cells were harvested and the fluorescence of cells was analyzed by a FCM device. The percentage of red fluorescent cells of HHRzm transfected cells was defined as 100%.

S1.6.3 Intracellular cleavage efficiency of HHRz variants target endogenous genes in cancer cells

Although TX-2 in all HHRz variants provided the best suppression of exogenous *mCherry* gene 48h post-transfection, we further tested the knockdown efficiency to target endogenous genes in MDA-MB-231 cells. Recombinant vectors containing HHRz, HHRzm and TX-2 were transfected into MDA-MB-231 cancer cells by Lipofactmin2000. 24h post-transfection, cells were collected and RNA expression of target genes were tested by RT-PCR (real-time RT-PCR). 48h post-transfection, cells were collected and protein expression of target genes were tested by WB (Western blot).

GAPDH encodes an enzyme which catalyzes the sixth step of glycolysis to play a role in glycolysis and nuclear functions. As house keeping genes, *GAPDH* is widely used to act as reference genes to normalize the expression of total RNA and protein. So we designed ribozymes to target *GAPDH*. As shown in Supplementary Figure S24, TX-2 down-regulated the expression of *GAPDH* in both mRNA and protein level more effectively than WT-HHRz. Then, we chose another gene to further confirm the knock-down efficiency of TX-2 in cancer cells. *BCL2* is a well-studied protooncogene which encodes a regulator protein to regulate cell death (apoptosis). Down-regulation of this gene in MDA-MB-231 cells leads to cellular growth inhibition. Therefore, we designed ribozymes to target *BCL2* to evaluate the intracellular *trans* RNA-cleaving efficiency of TX-2. As shown in Supplementary Figure S24, TX-2 also presented much better suppression of mRNA and protein expression of *BCL2* than WT-HHRz. Altogether, intracellular selected TX-2 displayed much better down-regulation efficiency in all targeted genes.

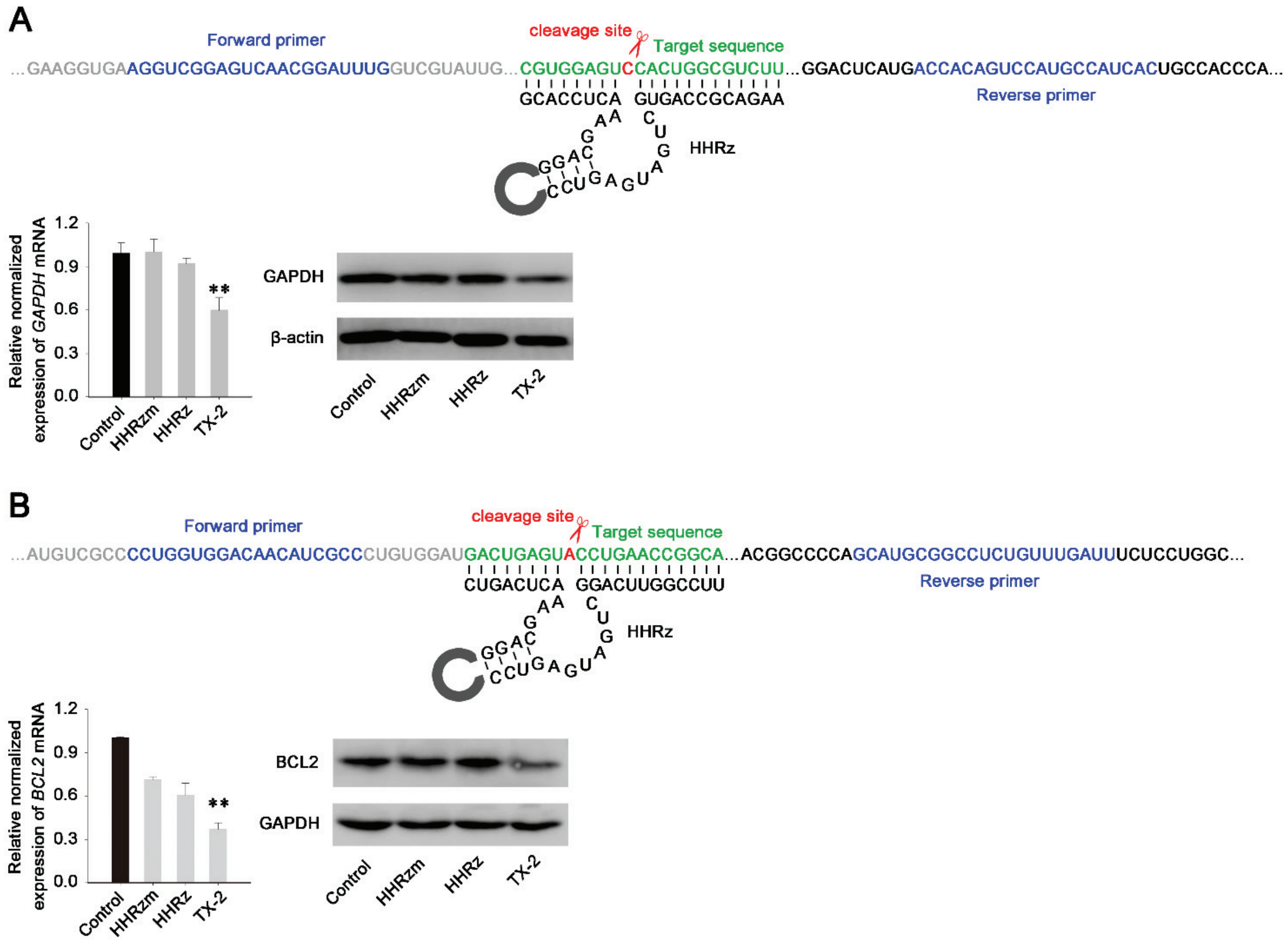

Supplementary Figure S24. Intracellular cleavage efficiency of HHRz variants target *GAPDH* (A) and *BCL2* (B) in MDA-MB-231 cells. The target sequences were labeled as green color and cleavage sites were labeled as red color; The locations of primer sequence for RT-PCR were highlighted by blue color. MDA-MB-231 cells were transfected with recombinant constructs containing HHRz, HHRzm and TX-2. At 24h post-transfection, cells were collected and the RNA expression of target genes were tested by RT-PCR. At 48h post-transfection, cells were harvested and the protein expression of target genes were analyzed by WB.

S1.8 Intracellular cleavage activity of HHRz variants in *zebrafish*

S1.7.1 Intracellular cleavage efficiency of HHRz variants target *mCherry* in *zebrafish*

The cleavage ability of selected mutants was further confirmed by suppression the same gene in fertilized *zebrafish* embryo. After 48 hours post fertilization, the red fluorescence of embryo injected by selected HHRz mutants are obliviously reduced compared with WT-HHRz. The reduction of mCherry expression leaded to most of the embryos containing selected HHRz mutants without visible red fluorescence, especially in TX2 injected embryo (Supplementary Figure S23).

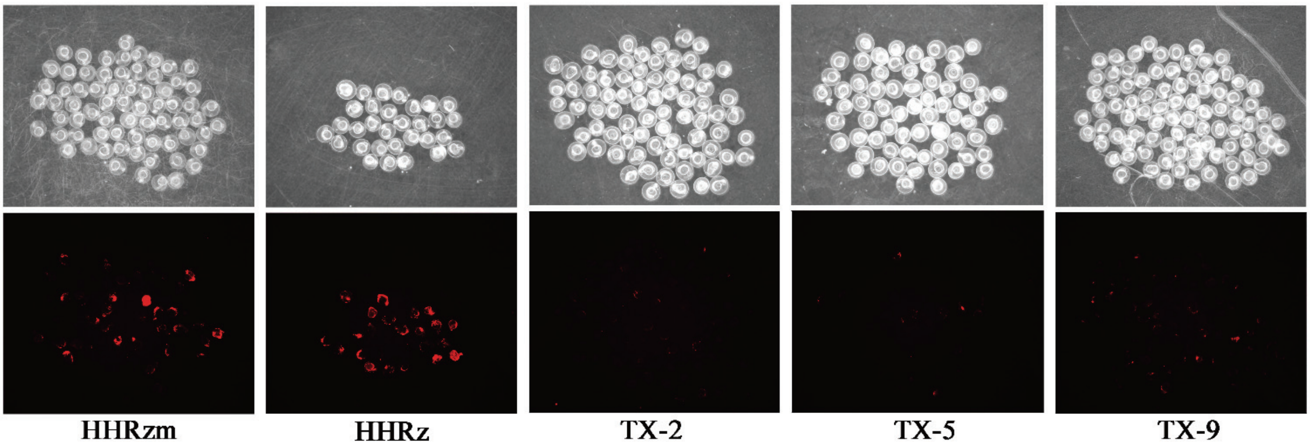

Supplementary Figure S25. Intracellular cleavage efficiency of HHRz variants target mCherry in *zebrafish*. 1 nl plasmid of each mutant targeted mCherry was microinjected into fertilized embryos. 48hpf, relative fluorescence intensity was confirmed by fluorescent microscope (sample size, HHRzm=67; HHRz=31; TX-2=76; TX-5=64; TX-9=85).

S1.7.2 Intracellular cleavage efficiency of HHRz variants target *nacre* in *zebrafish*

Another gene, *nacre*, was tested to evaluate the cleaved capacity of TX2 on endogenous locus. Similar phenotype modification of body pigmentation was observed as previous investigations (Supplementary Figure S24). The results of RT-PCR were consistent with the observation of phenotype modification. At 24 hours post fertilization (hpf), TX2 reduced the RNA expression of *nacre* more efficiently than WT HHRz (Figure 4B). These observations indicated that selected HHRz mutants especially TX2 could down-regulate expression of targeted protein in most cells of *Hela* and *zebrafish* embryo more effectively than WT HHRz.

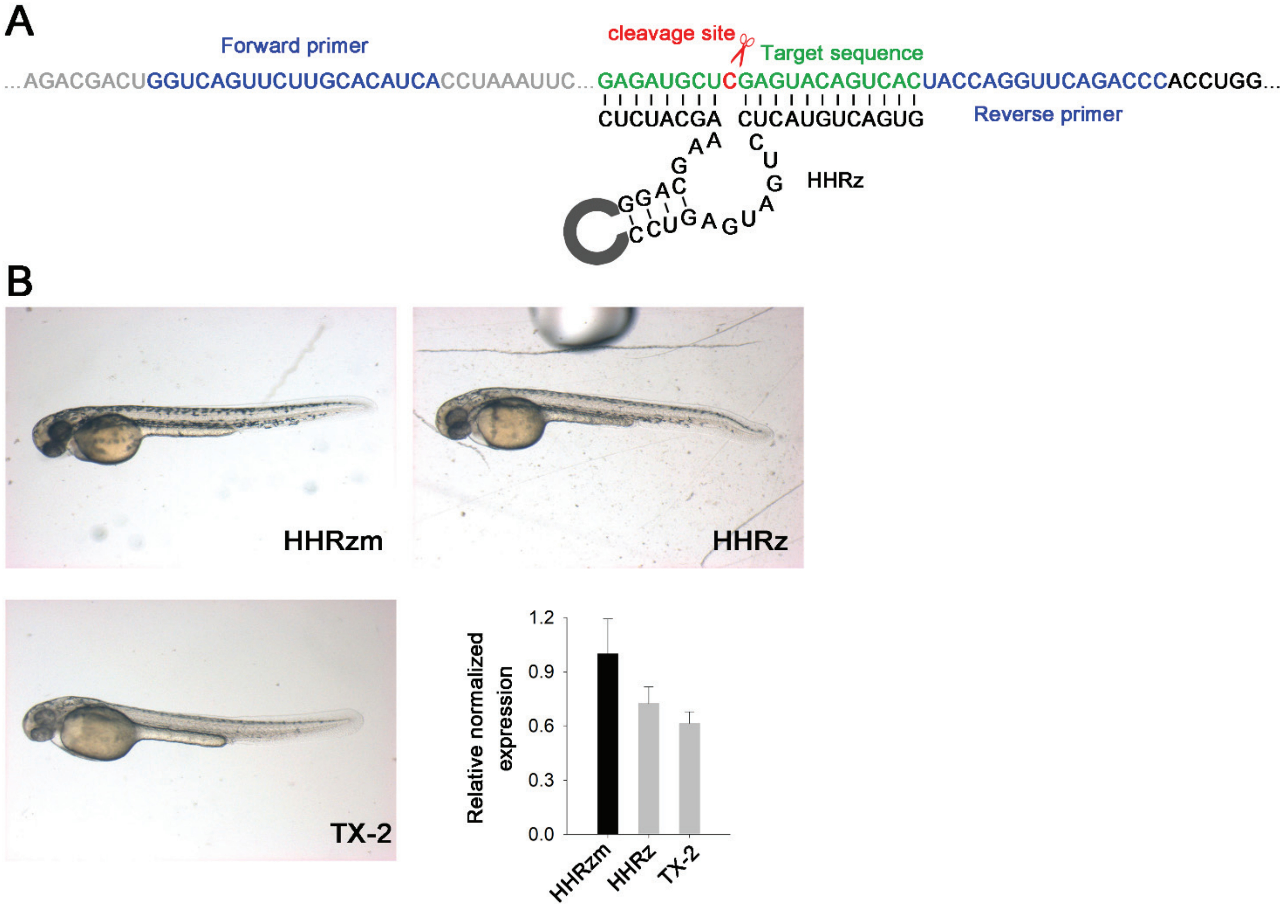

Supplementary Figure S26. Intracellular cleavage efficiency of HHRz variants target *nacre* in *zebrafish*. The target sequence was labeled as green color and cleavage site was labeled as red color; The locations of primer sequence for RT-PCR were highlighted by blue color. 1 nl plasmid of each mutant targeted nacre was microinjected into fertilized embryos. 50hpf, phenotype modifications were confirmed by fluorescent microscope (sample size, HHRzm=26; HHRz=46; TX-2=51). 24hpf, expression of *nacre* mRNA was detected by RT-PCR (sample size, HHRzm=36; HHRz=30; TX-2=39).

S1.7.2 Intracellular cleavage efficiency of HHRz variants target *ntl* in *zebrafish*

To confirm the selected HHRz variants could knock down genes as a general genetic manipulation tool in zebrafish, a well-characterized endogenous locus, *ntl/ntla*, was analyzed to assess the Intracellular performance of TX2. Previous studies have showed that zebrafish embryos homozygous for a null *ntl* mutation lack the notochord and tail. At 48 hpf, 37% embryos injected with ntl-targeted HHRz variants displayed typical no tail phenotype (Supplementary Figure S25). Real time polymerase chain reaction (RT-PCR) analysis revealed that TX2 induce down-regulation of ntl expression more efficiently than WT HHRz (Supplementary Figure S25). These results demonstrate that the TX2 is a good knockdown tool to investigate gene function in vertebrate embryonic development.

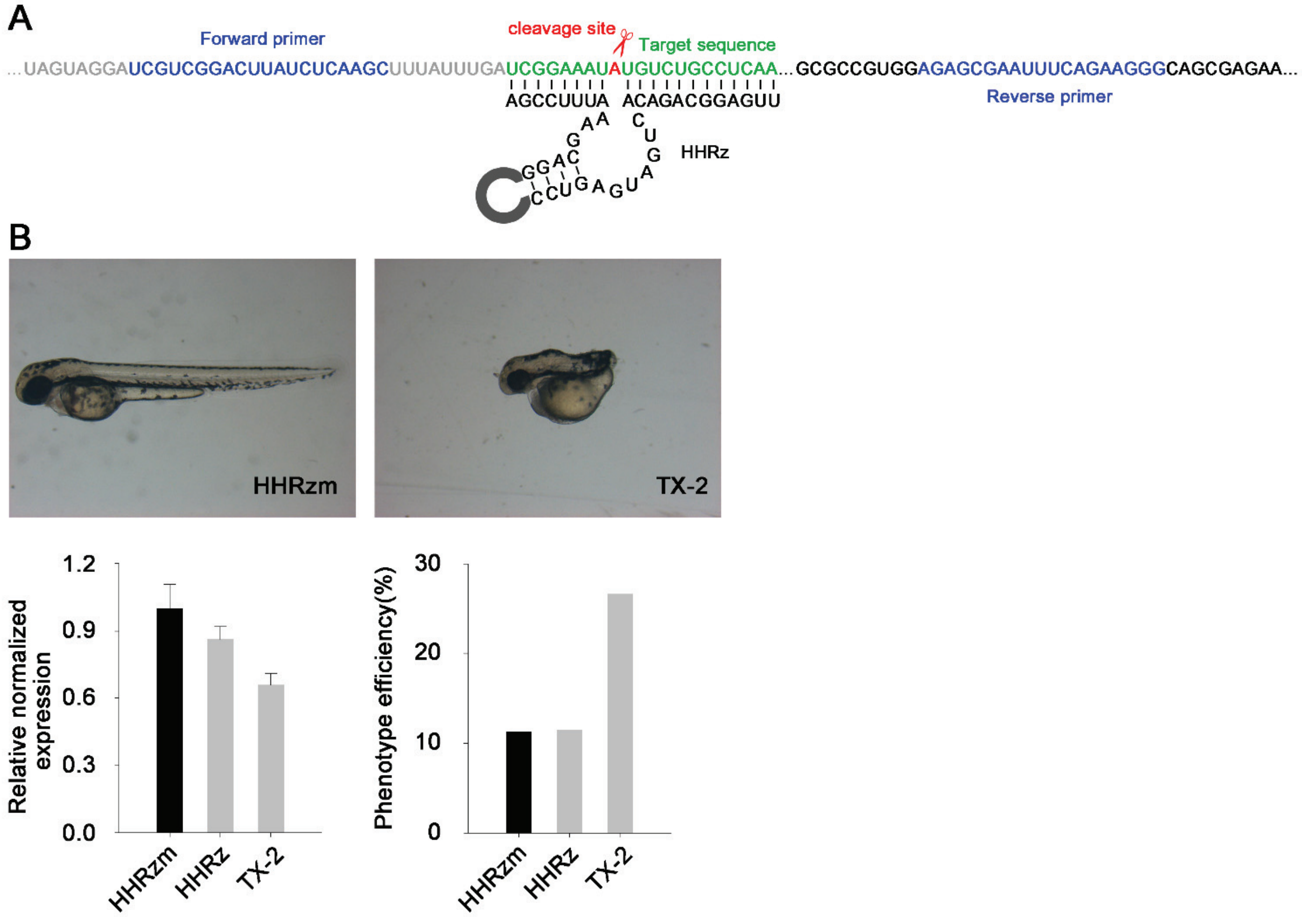

Supplementary Figure S27. Intracellular cleavage efficiency of HHRz variants target *ntl* in *zebrafish*. The target sequence was labeled as green color and cleavage site was labeled as red color; The locations of primer sequence for RT-PCR were highlighted by blue color. 1 nl plasmid of each mutant targeted *ntl* was microinjected into fertilized embryos. 28hpf, phenotype modifications were confirmed by fluorescent microscope (sample size, HHRzm=53; HHRz=26; TX-2=90). 24hpf, expression of *ntl* mRNA was detected by RT-PCR (sample size, HHRzm=43; HHRz=32; TX-2=35).

S2 Material and methods

S2.01 Library design and Synthesis of random single-strand DNA sequence

The DNA sequences of the selection pools for trans-acting HHRz were as follow:
R1, 5'-AACGAATTCCATGAATCCAG(N7)GTCCCAAATAGGACGAAACGCACCGAATCTAATACGGCCGCGGGTCCAGGGTTCAA GTCCCTGTTCGGGCGCCAGGATCCTCG-3'
R2, 5'-AACGAATTCCATGAATCCAGCTGATGAGTCC(N6)GGACGAAACGCACCGAATCTAATACGGCCGCGGGTCCAGGGTTCAA GTCCCTGTTCGGGCGCCAGGATCCTCG-3'.

N7 and N6 were 7 or 6 fully random nucleotides in long catalytic core and loop II of HHRz respectively. 5'-end of HHRz was restriction enzymes site (*Eco*RI), binding sequence of substrate and upstream linker connecting HHRz to tRNA; 3'-end of HHRz was downstream tRNA sequence, binding sequence of substrate and restriction enzymes site (*Bam*HI). X12 and X7 represent 12 or 8 nucleotides complementary with given sequence on substrate RNA. The selection dsDNA pools were prepared from fusion PCR products of ai-HR1/ai-HR2, ai-H2 and R-SR54. Fusion PCRs were performed as follows: pre-denaturation at 95 ºC for 1 min, then 10 cycles (30sec at 95 ºC, 30sec at 51 ºC, 40sec at 72 ºC of each cycle).

S2.02 Intracellular evolution of *trans*-cleaving HHRz by toxin protein

DNA products were purified by AxyPrepTM PCR cleanup Kit (Axygen) and double digested by *Eco*RI and *Bam*HI (NEB) in 37 ºC for 3h. Digested fragments of DNA pools were purified by AxyPrepTM PCR cleanup Kit (Axygen) again. DNA fragments of vector pTR7a1Gmai digested by same restriction enzyme were extracted by AxyPrepTM DNA Gel Extraction Kit (Axygen). Digested products were lined by NEB T4 ligase in 4ºC overnight giving raise of recombinant plasmid pools for intracellular selection. The presence of saline ions would decreas the transformed efficiency of recombinant vectors. The ligation products were extracted once with equal volume of phenol-chloroform extraction and then ethanol precipitated following recovery in 10μl sterile deionized water.

Electrotransformed competent cells were prepared according to the manufactories' protocol (Bio-Rad) with moderate modifications. Purified ligation products vectors were electrotransformed into bacterial expression strain BL21(DE3) and recovered in 37 ºC and 150 rpm for 1h. All cells were harvested by centrifugation in 4 ºC and 4000 rpm for 5 mins following coating on ampicillin resistant culture plate. In the induction of IPTG, cells with potential active HHRz mutants would survive on the plate and identified by DNA sequencing.

S2.03 Definition of *trans*-acting mutants based on dual fluorescent proteins using FCM

Recombinant vectors containing HHRz, HHRzm and selected variants were electrotransformed into bacterial expression strain BL21(DE3) and recovered in 37 ºC and 150 rpm for 1h. 50μl of transformed cells were coated on ampicillin resistant culture plate without IPTG. pTRDTa1GmaR-H with active HHRz was used as positive control and pTRDTa1GmaR-Hm with inactive HHRzm was construed as negative control.

More than three monoclones of each HHRz were inoculated in 1ml LB medium with ampicillin and cultivated in 37 ºC and 250 rpm until OD600 of culture medium reach 0.3. The cells in logarithmic phase were induced by IPTG (final concentration 1mM) in 37 ºC and 150 rpm for 18h. Then *E. coli* were centrifuged in 4 ºC and 4000 rpm for 5 mins and washed with PBS twice to abolish LB medium. Cell pellets were resuspended by PBS and analyzed by FCM (Green fluorescence, excitation at 480 nm, emission at 510nm; Red fluorescence, excitation 561 nm, emission 610 nm). EGFP were fusion expressed with mCherry and used as internal reference. Only cells with normal green fluorescence would be analyzed by FCM.

S2.04 Definition of *trans*-acting mutants variants via Spinach

Recombinant plasmids were electrotransformed into BL21(DE3) cells and cultivated on the resistant plate overnight. pTR7a1A3-H with active HHRz was used as positive control and pTR7a1A3-Hm with inactive HHRzm was construed as negative control. More than three monoclones of different HHRz mutants was picked up into 1ml LB medium with ampicillin in 37 ºC and 250 rpm. When the OD of newly inoculated bacteria liquor reached 0.3, final concentration 1 mM of IPTG was added into these medium to induce the expression of Spinach and HHRz mutants in 37 ºC and 150 rpm for 6h. All cells were centrifuged in 4 ºC and 4000 rpm for 5 mins. Cell pellets were

washed with 1 × PBS twice and resuspended with 500μl 1 × PBS. Resuspended cells were incubated with DFHBI (final concentration 400μl) at 37 ºC for 1h. Then the fluorescence of incubated cells with various HHRz variants were evaluated by FCM (exitation laser 488nm; emission, 510nm).

S2.05 *In vitro* transcription of RNA

Trans-cleaving HHRz sequence was amplified by PCR using the primers ICT7-1F, ICT7-2R, ai-H/ai-Hm/ai-XR2-2/ai-TX-2/ai-XR2-3/ai-XR2-5/ai-XR2-1/ai-TX-5/ai-XR2-4/ai-TX-9/ai-XR1-5/ai-XR1-4, ai-H2 or ai-H2m and R-SR54 (Supplementary Table 1). Cycling parameters of PCR were as follows: 95 ºC for 1 min, then 30 cycles of the following 30 sec at 95 ºC, 30 sec at 51 ºC, 40 sec at 72 ºC of each cycle). Substrate sequence of *trans*-cleaving HHRz was amplified using a primer including T7 promoter sequence in the 5' end of sequence (IRil-1Fa,5'-CGAAATTAATACGACTCACTATAGGGCTCGAGATTCGGTGCGTCCTGGATTCAATGATG-3') and a primer which binds downstream of IRil-1Fa (IRil-4R, 5'- AAGTCGCATCATTGAATCCAGGACGCACCG-3'). Fusion PCRs were performed for pre-denaturation at 95 ºC for 1 min, then 10 cycles (30 sec at 95 ºC, 30 sec at 51 ºC, 40 sec at 72 ºC of each cycle).

50μl PCR DNA product was used as template to transcribe *in vitro* at 37ºC using T7 RNA polymerase Kit from Fermentas following the manufacturer's instructions in a reaction mixture (160 μl) containing 32 μl 5× transcription Buffer, 25μl NTP mixture (10 mM), 10μl T7 RNA polymerase (20 U/μl) for 4h. Transcribed RNAs were precipitated by adding 0.1 volume of 3 M sodium acetate (pH 5.2) and 2 volume of water free ethanol in the presence of 20 mg glycogen. RNA was recovered by centrifugation at 20,000g for 15 min at 4ºC and washed twice with 100 μl 75% ethanol, then dried briefly under speedvac (Eppendorf, Cambridge, UK).

The pellet was resuspended in 10 μl Rnase free water. RNA was gel purified using 10% polyacrylamide gel. RNA was visualized by UV shadowing, excised, and eluted out by agitation in 500 μl Elution Buffer (0.01 M Tris-HCl, 0.2 M NaCl, 0.001 M EDTA) for 30min at RT temperature following centrifuging at 20,000g for 15 min at 4 ºC. The supernatant containing RNA was precipitated by adding 0.1 volume of 3 M sodium acetate (pH 5.2) and 2 volume of water free ethanol in the presence of 20 mg glycogen following drying under vacuum. The RNA was resuspended in 10 μl Rnase free water and quantitated by absorption at 260nm.

S2.06 p^{32} label of RNA substrate

Transcribed RNA substrates were treated with alkaline phosphatase to abolish the 5'-phosphate before the labeling experiment according to the manufacturer's suggestions. Briefly, RNA was incubated in a reaction mixture (10 μl) with FastAP (0.05 U/ml) in 10 mM Tris–HCl (pH 8.0), 5 mM $MgCl_2$, 100 mM KCl, 0.02% Triton X-100 and 0.1 mg/ml BSA at 37 ºC for 10 min. The mixture was extracted once with equal volume of phenol-chloroform extraction solution and then recovered by ethanol precipitation following drying under vacuum.

de-5'-phosphate RNA was resuspended in 10 μl Rnase free water. 1μl de-5'-phosphate RNA subtrate was incubated in a reaction mixture (10 μl) containing 50 mM Tris–HCl (pH 7.8), 40 mM NaCl,10 mM $MgCl_2$, 1 mg/ml BSA, 10 mCi[γ-32P] ATP and 10 units of PNK at 37 ºC for 1 h. The labeled product was purified by 10% denaturing PAGE.

S2.07 *In vitro* cleavage assay

The RNA of HHRz and target 5'-p32 labeled RNA was used for trans-cleavage reactions for 2h as described previously[7]. Briefly, excessive HHRz and target RNA substrate were incubated in a reaction mixture (10 μl) including 50 mM Tris–HCl (pH 8.4) and 10 mM $MgCl_2$. The mixture was heated to 65 ºC for 5 min, cooled to 37 ºC, and the *trans*-cleavage reaction was initiated by adding 20 mM $MgCl_2$. 2h later at 37 ºC, the reaction was terminated by addition 30 mM EDTA (final cencentration). Cleavage fragments were separated by 10% denaturing PAGE, and radioactivity was quantified using a phosphorimager (PerkinElmer Cyclone Plus Storage PhosphorSystem).

S2.08 Kinetic analysis of selected clones

Kinetic reaction contained 1 μM HHRz RNA, 150 nM 5'-p32 labelled RNA, 50 mM Tris–HCl (pH 7.4) and 0.5 mM $MgCl_2$ was incubated at 37 ºC for eight time points shown in the figure legends. At the indicated times, 5 μl of reaction were withdrawn and stopped by adding EDTA (pH 8.0) to a final concentration of 30 mM. The reaction products were mixed with 5 μl loading dye and RNA were analyzed by electrophoresis on a 10% polyacrylamide denaturing gel following quantifying by phosphorimager (PerkinElmer Cyclone Plus Storage PhosphorSystem).

Each HHRzs were conducted at least three independent experiments with each determination performed in triplicate. The experimental data was fit by non-linear regression analysis (exponential equation: Y=Ymax+(Y0-Ymax)×exp(-k_{obs}×X)). These experiments were performed at least in triplicate and the observed rate constant (k_{obs}) and maximum cleavage yield (CYmax) were determined.

S2.09 Multi-turnover Assay of Selected variants

Substrate sequence of multi-turnover assay was amplified using a primer including T7 promoter sequence (IRil-6F,5'-CGAAATTAATACGACTCACTATAGGGCTCGAGATTCGGTGCGTCCTGGATTCA-3') and a reverse complementary primer which binds downstream of IRil-6F (IRil-6R, 5'-TGAATCCAGGACGCACCG-3').

To detect the rate of the HHRz mutants in multiple turnover conditions, 10 nM RNA of HHRz mutants with a 10-fold excess of substrate was incubated at 37ºC for 6h in a reaction buffer containing 50 mM Tris–HCl (pH 7.4) and 0.5 mM $MgCl_2$. 6h later, the reaction products were mixed with 5 μl loading dye and RNA were analyzed by electrophoresis on a 10% polyacrylamide denaturing gel following quantifying by phosphorimager (PerkinElmer Cyclone Plus Storage PhosphorSystem). These experiments were performed at least in triplicate and data shown are representative of three independent experiments.

S2.10 Intracellular cleavage activity of selected HHRz variants target mCherry in Hela cells

One day before transfection, 1×10^6 *Hela* cells were inoculated in each well of six well plate. *Hela* cells were transfected with 5 μg of recombinant constructs containing HHRz, HHRzm and variants for 5 h using Lipofectamine2000 (Invitrogen) according to the manufacturer's instructions. 48 h post-transfection, cells were harvested and washed twice by PBS. Cells were resuspended in 1000 μl PBS, then analyzed on a FCM device (Moflo XDP, Beckman Coulter).

S2.11 Intracellular cleavage activity of selected HHRz variants target other genes in cancer cells

One day before transfection, 0.7×10^6 MDA-MB-231 cells were inoculated in each well of six well plate. MDA-MB-231 cells were transfected with 2.5 μg of recombinant constructs containing HHRz, HHRzm and variants for 5 h using Lipofectamine2000 (Invitrogen) according to the manufacturer's instructions. 24h post-transfection, cells were harvested for real time polymerase chain reaction (RT-PCR). 48 h post-transfection, cells were harvested for Western blotting (WB).

S2.12 RT-PCR of cancer genes

24 h post-transfection, cells were harvested and lysed in TRIzol Reagent (Invitrogen). Total RNA was extracted according to the manufacturer's instruction and stored at -20°C. DNA was synthesized from 1 μg of RNA using TransScript One-Step gDNA Removal and cDNA Synthesis SuperMix Kit (Transgen) according to the manufacturer's instructions. Synthesized cDNA was stored at -20 °C. Quantitative Real-Time PCR was carried out in triplicate using SYBR green on a real-time PCR detection system (Bio-Rad CFX Connect, CA, USA). All the primers of RT-PCR were designed to span the cleavage site. For each gene, gene expression levels were calculated relative to a reference gene, *GAPDH or ACTB,* which was frequently-used to normalize the level of the total mRNA expression.

The primers used for real time PCR are listed as below:

gene	primer	
BCL2	Forward primer	5'-CCTGGTGGACAACATCGCC-3'
	Reverse primer	5'-AATCAAACAGAGGCCGCATGC-3'
GAPDH	Forward primer	5'-AGGTCGGAGTCAACGGATTTG-3'
	Reverse primer	5'-GTGATGGCATGGACTGTGGT-3'
ACTB	Forward primer	5'-CCTCGCCTTTGCCGATCC-3'
	Reverse primer	5'-GGATCTTCATGAGGTAGTCAGTC-3'

Cycling parameters of genes were as follows: 95 °C × 3 min, then 40 cycles of the following 95 ºC 10 sec, 58 ºC 30 sec, 72 ºC 30 sec.

Data generated by RT-PCR were compiled and collected using CFX Manager 3.1 software (Bio-Rad). Each experiment was performed at least in duplicate, using independent biological samples.

S2.13 Western blotting (WB) of cancer genes

Whole-cell protein extracts were prepared using RIPA lysis buffer (PC101, EpiZyme, CHINA) and detected by Western blot analysis. Antibodies used were rabbit monoclonal to GAPDH (ab128915, Abcam, Cambridge, UK), rabbit monoclonal to Bcl-2 (ab32124, Abcam, Cambridge, UK) and rabbit monoclonal to β-Actin (ab115777, Abcam, Cambridge, UK). Proteins recognized by the antibodies were detected by ImageQuant LAS 500 (GE, CT, USA) using HRP-conjugated Goat Anti-Rabbit secondary antibody (BBI, Sangon Biotech, CHINA).

S2.14 Intracellular cleavage activity of selected HHRz variants in *zebrafish*

WT adult male and female *zebrafish*, *Danio rerio*, were maintained in 30 gal aquaria at 28 °C on a 14:10 light-dark cycle. Fertilized embryos were obtained after natural spawning, washed, distributed into 20 × 100 mm culture plates and maintained at 28.5 °C. AB strains were used in our studies. All *zebrafih* experiments were performed in accordance with the guidelines of the animal ethical committee of West China Hospital. All experimental protocols were proved by the Animal Ethical Committee, West China Hospital, Sichuan University.

Recombinant vectors were prepared by AxyPrep Endo-Free Miniprep kit (Axygen) and quantified by NanoDrop Microvolume Spectrophotometers and Fluorometer (thermofisher). The plasmids were injected into the yolk at the one cell stage. Unless stated otherwise, a volume of 1 nl was injected into embryos with the concentration of 300 ng/μl of plasmid. For morphological assessment, embryos were raised to 24–30 hpf and imaged, with one exception, nacre were raised to 50 hpf and imaged by microscope (ZEISS Axio Zoom.V16).

S2.15 RT-PCR of *zebrafish* genes

24hpf, all the embryos and tissues were homogenized and frozen in TRIzol Reagent (Invitrogen) and stored at -80 °C. Total RNA was extracted according to the manufacturer's instruction. cDNA was synthesized from 1 μg of RNA with oligo(dT) priming using TaKaRa PrimeScript TM RT reagent Kit according to the manufacturer's instructions. Synthesized cDNA was stored at -20 °C. Quantitative Real-Time PCR was carried out in triplicate using the Roche FastStart Essential DNA Green Master on a real-time PCR detection system (Bio-Rad CFX96). All the primers of RT-PCR were designed to span the cleavage site. For each gene, gene expression levels were calculated relative to a reference gene, *gapdh*.

The primers used for real time PCR are listed as below:

gene	primer	
ntl	Forward primer	5'-TCGTCGGACTTATCTCAAGC-3'
	Reverse primer	5'-CCCTTCTGAAATTCGCTCT-3'
nacre	Forward primer	5'-GGTCAGTTCTTGCACATCA-3'
	Reverse primer	5'-GGGTCTGAACCTGGTAGTG-3'
gapdh	Forward primer	5'-ACCCGTGCTGCTTTCTTGAC-3'
	Reverse primer	5'-GACCAGTTTGCCGCCTTCT-3'

Cycling parameters of *ntl* gene were as follows: 95 °C × 3 min, then 40 cycles of the following 95 ºC 10 sec, 61 ºC 20 sec, 72 ºC 20 sec. For *nacre* gene, cycling parameters were as follows: 95 °C × 3 min, then 40 cycles of the following 95 ºC 10 sec, 62 ºC 20 sec, 72 ºC 20 sec.

Knockdown efficiencies were calculated as the ratio of normalized gene expression in plasmid-injected versus uninjected sample. Data generated by real-time PCR were compiled and collected using CFX Manager 3.1 software (Bio-Rad). Each experiment was performed at least in duplicate, using independent biological samples.

S3.Supplementary Table S1. Primers of plasmid construction and *in vitro* transcription

Primer	Sequence
ibsC-5H	GTCAAGCTTATGATGCGACTTGTCATCATACTGATTGTACTGTTACTCATAAGTTTCAG
ibsC-3X	GCTCTCGAGTTAATAAGCGCTGAAACTTATGAGTAACAGTACAATCAGTATGATGACAA
RBS5	GCTTCTAGAAATAATTTTGTTTAACTTTAAGAAGGAGATATACATATGGGATCCTCG
RBS3	CGAGGATCCCATATGTATATCTCCTTCTTAAAGTTAAACAAAATTATTTCTAGAAGC
BH-5	GCAGGATCCGAGCTCCGTCGTTACAAGCTTTCG
BH-3	CGAAAGCTTGTAACGACGGAGCTCGGATCCTGC
EI5	TGGCGCCGGTGATGCCGGCCACGATGCGT
Tac2	GATGCCGGCCACGATGCGTCCGGCGTAGAGGATCGAGATCGATCTCGATCCCGCGAAAT
TaR2c	CATTATACGAGCCGGATGATTAATTGTCAAATTTCGCGGGATCGAGATCG
Tac3R	GCTTCTAGAGGCCCATTATACGAGCCGG
HHRza5	GCTGGATCCCAGGTACATCCAGCTGATGAGTCCCAAATAGGACGAAACGCGCTTCGG
HHRza5m	GCTGGATCCCAGGTACATCCAGCTGATGAGTCCCAAATAGGACGAGACGCGCTTCGG
HHRza3	CGAAAGCTTCGCAGTGGAATCCAGGACGCACCGAAGCGCGTTTCGTCC
HHRza3m	CGAAAGCTTCGCAGTGGAATCCAGGACGCACCGAAGCGCGTCTCGTCC
RzaS5m4	CGAAATTAATACGACTCACTATAGGGACTGTTGTGCGTCCTGGATTCCACTGCTCAG
RzaS3a	GCAGTGGAATCCAGGACGCACCCCTATAGTGAGTCGTATTAATTTCG
RzaF1	CGAAATTAATACGACTCACTATAGGGAGCGCTGAGCAGGTACATCCAG
Rza-sR1	ACTGTTGCGCGTTTCGTCCTATTTGGGACTCATCAGCTGGATGTACCTGCTCAGCGCT
Rzam-sR1	ACTGTTGCGCGTGTCGTCCTATTTGGGACTCATCAGCTGGATGTACCTGCTCAGCGCT
T7TFS	ATAGGATCCAGCATAACCCCTTGGG
T7TFL.1	AGCATAACCCCTTGGGGCCTCTAAACGGGTCTTGAGGGGTTTTTTGTGCTTAAGTTGTACACGGCCGCA
iT7-1b	ATCCGCTCACAATTCCCCTATAGTGAGTCGTATTAATTTCGATTATGCGGCCGTGTACA
iT7-2bR	CTTAAAGTTAAACAAAATTATTGGGGAATTGTTATCCGCTCACAATTCCCC
iT7-3aR	CATAAGCTTCATATGTATATCTCCTTCTTAAAGTTAAACAAAATTATTGGGGAATTGTT
12/10z	GCTTCTAGAGCCCGGATAGCTCAGTCGGTAGAGCAGCGGCCGTACTTCCACCAACGAAT
12/10zR	CGTGAATTCGTTGGTGGAAGTACGGCCGCTGCTCTACCGACTGAGCTATCCGGGCTCTA
ai-H1	ACTTCCACCAACGAATTCCATGAATCCAG
ai-H	AACGAATTCCATGAATCCAGCTGATGAGTCCCAAATAGGACGAAACGCACCG
ai-Hm	AACGAATTCCATGAATCCAGCTGATGAGTCCCAAATAGGACGAGACGCACCG
ai-HR1	AACGAATTCCATGAATCCAGNNNNNNNGTCCCAAATAGGACGAAACGCACCG
ai-HR2	AACGAATTCCATGAATCCAGCTGATGAGTCCNNNNNNGGACGAAACGCACCG
ai-H2	GGACGAAACGCACCGAATCTAATACGGCCGCGGGTC
ai-H2m	GGACGAGACGCACCGAATCTAATACGGCCGCGGGTC
R-SR54	CGAGGATCCTGGCGCCCGAACAGGGACTTGAACCCTGGACCCGCGGCCGTATTA
T7X	GCTTCTAGAGGCCTATAGTGAGTCGTATTAATTTCGCGGGATCGAGATCG
GFP-5H	CTAAAGCTTATGGTGAGCAAGG
GFP-3mX	GCGCTCGAGCTTGTACAGCTCGTCCATGC
ibsCb-5X	GTCCTCGAGATTCGGTGCGTCCTGGATTCAATGATGCGACTTGTCATCATACTGATTGT
ibsC-3B	GCTGCTCAGCTTAATAAGCGCTGAAACTTATGAGTAACAGTACAATCAGTATGATGACA
IRil-1Fa	CGAAATTAATACGACTCACTATAGGGCTCGAGATTCGGTGCGTCCTGGATTCAATGATG
IRil-4R	AAGTCGCATCATTGAATCCAGGACGCACCG
ICT7-1F	CGAAATTAATACGACTCACTATAGGGCTCTAGAGCCCGGATAGCTCAGTCGGTAGAGCA
ICT7-2R	CTGGATTCATGGAATTCGTTGGTGGAAGTACGGCCGCTGCTCTACCGACTGAG
ai-XR2-2	AACGAATTCCATGAATCCAGCTGATGAGTCCGCCTTCGGACGAAACGCACCG
ai-TX-2	AACGAATTCCATGAATCCAGCTGATGAGTCCGGTAGCGGACGAAACGCACCG
ai-XR2-3	AACGAATTCCATGAATCCAGCTGATGAGTCCCGTCGTGGACGAAACGCACCG
ai-XR2-5	AACGAATTCCATGAATCCAGCTGATGAGTCCCTACGGGGACGAAACGCACCG
ai-XR2-1	AACGAATTCCATGAATCCAGCTGATGAGTCCTAATGCGGACGAAACGCACCG
ai-TX-5	AACGAATTCCATGAATCCAGCTGATGAGTCCAGTGCTGGACGAAACGCACCG
ai-XR2-4	AACGAATTCCATGAATCCAGCTGATGAGTCCGCTGGAGGACGAAACGCACCG
ai-TX-9	AACGAATTCCATGAATCCAGCTGATGAGTCCGAGGACGGACGAAACGCACCG
ai-XR1-5	AACGAATTCCATGAATCCAGCGCTTAGGTCCCAAATAGGACGAAACGCACCG
ai-XR1-4	AACGAATTCCATGAATCCAGCGAACGGGTCCCAAATAGGACGAAACGCACCG
RI-5X	GCACTCGAGATTCGGTGCGTCCTGGATTCAATGGTGAGCAAGGGCGAGGAGGATAAC
R-3B	GCTGCTGAGCCTACTTGTACAGCTCGTC
iTac-1R	GTTCGCATTATACGAGCCGGATGATTAATTGTCAAATTTCGATTATGCGGCCGTGTACA
iTac-2	CGTATAATGCGAACAGAAAGTTTGACAATTAATCATCCGGCTCGTATAATGGGGGAATT
iTac-3R	ATTATTGGGGAATTGTTATCCGCTCACAATTCCCCCATTATAC
R260SaH	AACGAATTCCAGACAGCTTCAAGCTGATGAGTCCCAAATAGGACGAAAGTCGGGGTAAT
R260SaTX-2	AACGAATTCCAGACAGCTTCAAGCTGATGAGTCCGGTAGCGGACGAAAGTCGGGGTAAT
R260SaTX-5	AACGAATTCCAGACAGCTTCAAGCTGATGAGTCCAGTGCTGGACGAAAGTCGGGGTAAT
R260SaTX-9	AACGAATTCCAGACAGCTTCAAGCTGATGAGTCCGAGGACGGACGAAAGTCGGGGTAAT
R260SaHm	AACGAATTCCAGACAGCTTCAAGCTGATGAGTCCCAAATAGGACGAGAGTCGGGGTAAT
R260Sb	GGACGAAAGTCGGGGTAATACGGCCGCGGGTC
R374SaH	AACGAATTCCAAGCTTCACCTTGCTGATGAGTCCCAAATAGGACGAAAGATGAACTAAT
R374SaHm	AACGAATTCCAAGCTTCACCTTGCTGATGAGTCCCAAATAGGACGAGAGATGAACTAAT

R374Sb	GGACGAAAGATGAACTAATACGGCCGCGGGTC
R467SaH	AACGAATTCCACCGTCCTCGGGGCTGATGAGTCCCAAATAGGACGAAACATCCGCTAAT
R467SaHm	AACGAATTCCACCGTCCTCGGGGCTGATGAGTCCCAAATAGGACGAGACATCCGCTAAT
R467Sb	GGACGAAACATCCGCTAATACGGCCGCGGGTC
R533SaH	AACGAATTCCAACCTCAGCGTCGCTGATGAGTCCCAAATAGGACGAAAGTGGCCGTAAT
R533SaTX-2	AACGAATTCCAACCTCAGCGTCGCTGATGAGTCCGGTAGCGGACGAAAGTGGCCGTAAT
R533SaTX-5	AACGAATTCCAACCTCAGCGTCGCTGATGAGTCCAGTGCTGGACGAAAGTGGCCGTAAT
R533SaTX-9	AACGAATTCCAACCTCAGCGTCGCTGATGAGTCCGAGGACGGACGAAAGTGGCCGTAAT
R533SaHm	AACGAATTCCAACCTCAGCGTCGCTGATGAGTCCCAAATAGGACGAGAGTGGCCGTAAT
R533Sb	GGACGAAAGTGGCCGTAATACGGCCGCGGGTC
tA-5H	GCTAAGCTTGCCCGGATAGCTCAGTCGGTAGAGCAGCGG
tA-3X	CGTCTCGAGTGGCGCCCGAACAGGGACTTGAACCCTGGACCCGCGG
tA-2	CGGTAGAGCAGCGGGACGCGACCGAAATGGTGAAGGACGGGTCCAGTGCGTACTGTTAC
tA-3	GTGCGTACTGTTACTCATAAGTTTCAGCGCTGCACTGTTGAGTAGAGTGTGAGCTCCGT
tA-4R	CCCTGGACCCGCGGGACGCGACCAGTTACGGAGCTCACACT
tA-2a	GGTAGAGCAGCGGGACGCGACCGAAATGGTGAAGGACGGGTCCAGTGCaGTACTGTTAC
tA-3a	TGCaGTACTGTTACTCATAAGTTTCAGCGCTtGCACTGTTGAGTAGAGTGTGAGCTCCG
tA-2b	GGTAGAGCAGCGGGACGCGACCGAAATGGTGAAGGACGGGTCCAGTGCatGTACTGTTA
tA-3b	GCatGTACTGTTACTCATAAGTTTCAGCGCTatGCACTGTTGAGTAGAGTGTGAGCTCC
tA-5	CGGTAGAGCAGCGGCCGTACTTGTACTGTTACTCATAAGTTTCAGCGCTCCACCAACAA
tA-6	CGCTCCACCAACAAAATCCAGACGCGACCGAAATGGTGAAGGACGGGTCCAGTGCTTCG
tA-7R	ATTAGACGCGACCAGTTACGGAGCTCACACTCTACTCAACAGTGCCGAAGCACTGGACC
tA-8R	CCCTGGACCCGCGGCCGTATTAGACGCGACCA
AD7-2R	CGTAAGCTTGGGGAATTGTTATCCGCTCACAATTCC
i42-H1	AACGAATTCCAGCGCTGAAACT
i42-H	CCAGCGCTGAAACTCTGATGAGTCCCAAATAGGACGAAATGAGTAATAATACGGCCGCG
i42-Hm	CCAGCGCTGAAACTCTGATGAGTCCCAAATAGGACGAGATGAGTAATAATACGGCCGCG
i42-TX-2	CCAGCGCTGAAACTCTGATGAGTCCGGTAGCGGACGAAATGAGTAATAATACGGCCGCG
i42-TX-5	CCAGCGCTGAAACTCTGATGAGTCCAGTGCTGGACGAAATGAGTAATAATACGGCCGCG
i42-TX-9	CCAGCGCTGAAACTCTGATGAGTCCGAGGACGGACGAAATGAGTAATAATACGGCCGCG
i42-H2R	CGAGGATCCTGGCGCCCGAACAGGGACTTGAACCCTGGACCCGCGGCCGTATTATTAC
IRil-6F	CGAAATTAATACGACTCACTATAGGGCTCGAGATTCGGTGCGTCCTGGATTCA
IRil-6R	TGAATCCAGGACGCACCG
t1F	CTAGTCGACGCCCGGATAGCTCAGTCGGTAGAGCAGCGGCCGTA
t2R	TGGAATTCGTTGGTGGAAGTACGGCCGCTGCTC
Rt260F1H	AACGAATTCCAGACAGCTTCAAGCTGATGAGTCCCAAATAGGACGAAAGTCGGGGTAAT
Rt260F1Hm	AACGAATTCCAGACAGCTTCAAGAAAAAAAGTCCCAAATAGGACGAAAGTCGGGGTAAT
Rt260F1X2	AACGAATTCCAGACAGCTTCAAGCTGATGAGTCCGGTAGCGGACGAAAGTCGGGGTAAT
Rt260F1X5	AACGAATTCCAGACAGCTTCAAGCTGATGAGTCCAGTGCTGGACGAAAGTCGGGGTAAT
Rt260F1X9	AACGAATTCCAGACAGCTTCAAGCTGATGAGTCCGAGGACGGACGAAAGTCGGGGTAAT
Rt260F2	GGACGAAAGTCGGGGTAATACGGCCGCGGGTC
Lt1032F1H	AACGAATTCCATGCCGGTTCAGGCTGATGAGTCCCAAATAGGACGAAACTCAGTCTAAT
Lt1032F1Hm	AACGAATTCCATGCCGGTTCAGGAAAAAAAGTCCCAAATAGGACGAAACTCAGTCTAAT
Lt1032F1X2	AACGAATTCCATGCCGGTTCAGGCTGATGAGTCCGGTAGCGGACGAAACTCAGTCTAAT
Lt1032F2	GGACGAAACTCAGTCTAATACGGCCGCGGGTC
Gt369F1H	AACGAATTCCAAAGACGCCAGTGCTGATGAGTCCCAAATAGGACGAAACTCCACGTAAT
Gt369F1Hm	AACGAATTCCAAAGACGCCAGTGAAAAAAAGTCCCAAATAGGACGAAACTCCACGTAAT
Gt369F1X2	AACGAATTCCAAAGACGCCAGTGCTGATGAGTCCGGTAGCGGACGAAACTCCACGTAAT
Gt369F2	GGACGAAACTCCACGTAATACGGCCGCGGGTC
RtH-4R	GCTAAGCTTAAAAAATGGCGCCCGAACAGGGACTTGAACCCTGGACCCGCGGCCGTATT
bGH -5X	CTGCAGAACTCGAGCTTAACTGTGCCTTCTAGTTGCCAGCCAT
bGH -3H	GCTAAGCTTCCATAGAGCCCACCGCATCCCCAG
U6F1-B	GCAGGATCCAAGGTCGGGCAGGAAGAGGGCCTATTTCCCATGATTCCTTCATATTTGCA
U6L1	GATTCCTTCATATTTGCATATACGATACAAGGCTGTTAGAGAGATAATTAGAATTAATTTGACTGTAAACACAAAGATATTAG
U6L2	GTAAACACAAAGATATTAGTACAAAATACGTGACGTAGAAAGTAATAATTTCTTGGGTAGTTTGCAGTTTTTAAAATTATG
U6L3R	GATATATAAAGCCAAGAAATCGAAATACTTTCAAGTTACGGTAAGCATATGATAGTCCATTTTAAAACATAATTTTAAAAACTGC
U6L4R-S	AAGGTCGACTAGTATATGTGCTGCCGAAGCGAGCACGGTGTTTCGTCCTTTCCACAAGATATATAAAGCCAAGAA
NtH95F2	GGACGAAAGCATCTCTAATACGGCCGCGGGTC
NtH95F1H	AACGAATTCCAGTGACTGTACTCCTGATGAGTCCCAAATAGGACGAAAGCATCTCTAAT
NtH95F1Hm	AACGAATTCCAGTGACTGTACTCAAAAAAAGTCCCAAATAGGACGAAAGCATCTCTAAT
NtH95F1X2	AACGAATTCCAGTGACTGTACTCCTGATGAGTCCGGTAGCGGACGAAAGCATCTCTAAT
NtH95F1X5	AACGAATTCCAGTGACTGTACTCCTGATGAGTCCAGTGCTGGACGAAAGCATCTCTAAT
NtH95F1X9	AACGAATTCCAGTGACTGTACTCCTGATGAGTCCGAGGACGGACGAAAGCATCTCTAAT
Tt84F2	GGACGAAATTTCCGATAATACGGCCGCGGGTC
Tt84F1H	AACGAATTCCATTGAGGCAGACACTGATGAGTCCCAAATAGGACGAAATTTCCGATAAT
Tt84F1Hm	AACGAATTCCATTGAGGCAGACAAAAAAAAGTCCCAAATAGGACGAAATTTCCGATAAT
Tt84F1X2	AACGAATTCCATTGAGGCAGACACTGATGAGTCCGGTAGCGGACGAAATTTCCGATAAT
Tt84F1X9	AACGAATTCCATTGAGGCAGACACTGATGAGTCCGAGGACGGACGAAATTTCCGATAAT

Tt84F1X5	AACGAATTCCATTGAGGCAGACACTGATGAGTCCAGTGCTGGACGAAATTTCCGATAAT

S4.Supplementary Table S2. Plasmids used in the work

Plasmid	Source	Notes
pET32		
pmCherry-C1		
pcDNA3.1		
pTaOi	This work	Tac promoter to initiate the expression of HHRz
pT7Oi	This work	T7 promoter to initiate the expression of HHRz, without HHRz
pT7Oi-HHRz	This work	T7 promoter to initiate the expression of HHRz, with active HHRz
pT7Oi-HHRzm	This work	T7 promoter to initiate the expression of HHRz, with inactive HHRz
pTR7a1Gmai-H	This work	Two T7 promoter to initiate the expression of HHRz and ibsC independently
pTR7a1Gmai-Hm	This work	Two T7 promoter to initiate the expression of HHRz and ibsC independently
pTRDTa1GmaR-H	This work	T7 promoter and tandem dual Tac promoter to initiate the expression HHRz, and EGFP plus mCherry independently
pTRDTa1GmaR-Hm	This work	
pTRDTa1GmaR260H	This work	
pTRDTa1GmaR260Hm	This work	
pTRDTa1GmaR374H	This work	
pTRDTa1GmaR374Hm	This work	
pTRDTa1GmaR467H	This work	
pTRDTa1GmaR467Hm	This work	
pTRDTa1GmaR533H	This work	
pTRDTa1GmaR533Hm	This work	
pTR7a1A1-H	This work	Two T7 promoter to initiate the expression of HHRz and Spinach independently
pTR7a1A1-Hm	This work	
pTR7a1A2-H	This work	
pTR7a1A2-Hm	This work	
pTR7a1A3-H	This work	
pTR7a1A3-Hm	This work	
pTR7a1A4-H	This work	
pTR7a1A4-Hm	This work	
pRUT-Rt260H	This work	CMV and U6 to initiate the expression of HHRz and mCherry independently
pRUT-Rt260Hm	This work	
pRUT-Lt1032H	This work	
pRUT-Lt1032Hm	This work	
pRUT-Gt369H	This work	
pRUT-Gt369Hm	This work	
pRUT-Nt95H	This work	
pRUT-Nt95Hm	This work	
pRUT-Tt84H	This work	
pRUT-Tt84Hm	This work	

References

1. Fozo, E.M. et al. Repression of small toxic protein synthesis by the Sib and OhsC small RNAs. *Mol Microbiol* **70**, 1076-93 (2008).
2. Ponchon, L. & Dardel, F. Recombinant RNA technology: the tRNA scaffold. *Nat Methods* **4**, 571-6 (2007).
3. Medina, M.F. & Joshi, S. Design, characterization and testing of tRNA3Lys-based hammerhead ribozymes. *Nucleic Acids Res* **27**, 1698-708 (1999).
4. Paige, J.S., Wu, K.Y. & Jaffrey, S.R. RNA mimics of green fluorescent protein. *Science* **333**, 642-6 (2011).
5. Nakayama, S., Luo, Y., Zhou, J., Dayie, T.K. & Sintim, H.O. Nanomolar fluorescent detection of c-di-GMP using a modular aptamer strategy. *Chem Commun (Camb)* **48**, 9059-61 (2012).
6. Paige, J.S., Nguyen-Duc, T., Song, W. & Jaffrey, S.R. Fluorescence imaging of cellular metabolites with RNA. *Science* **335**, 1194 (2012).
7. Ramezani, A. & Joshi, S. Comparative analysis of five highly conserved target sites within the HIV-1 RNA for their susceptibility to hammerhead ribozyme-mediated cleavage in vitro and in vivo. *Antisense Nucleic Acid Drug Dev* **6**, 229-35 (1996).
